# Clinical determinants and neural correlates of presbyphagia in community-dwelling older adults

**DOI:** 10.3389/fnagi.2022.912691

**Published:** 2022-07-28

**Authors:** Bendix Labeit, Paul Muhle, Jonas von Itter, Janna Slavik, Andreas Wollbrink, Peter Sporns, Thilo Rusche, Tobias Ruck, Anna Hüsing-Kabar, Reinhold Gellner, Joachim Gross, Rainer Wirth, Inga Claus, Tobias Warnecke, Rainer Dziewas, Sonja Suntrup-Krueger

**Affiliations:** ^1^Department of Neurology, Institute of Translational Neurology, University Hospital Münster, Münster, Germany; ^2^Institute for Biomagnetism and Biosignal Analysis, University Hospital Münster, Münster, Germany; ^3^Department of Neuroradiology, Clinic for Radiology & Nuclear Medicine, University Hospital Basel, Basel, Switzerland; ^4^Department of Diagnostic and Interventional Neuroradiology, University Medical Center Hamburg-Eppendorf, Hamburg, Germany; ^5^Department of Neurology, Heinrich Heine University Düsseldorf, Düsseldorf, Germany; ^6^Medical Clinic B (Gastroenterology, Hepatology, Endocrinology and Clinical Infectiology), University Hospital Münster, Münster, Germany; ^7^Department of Geriatric Medicine, Marien Hospital Herne, Herne, Germany; ^8^Department of Neurology and Neurorehabilitation, Hospital Osnabrück, Osnabrück, Germany

**Keywords:** oropharyngeal dysphagia, geriatrics, malnutrition, sarcopenia, neuroimaging, presbyphagia, neuroscience

## Abstract

**Background:**

“Presbyphagia” refers to characteristic age-related changes in the complex neuromuscular swallowing mechanism. It has been hypothesized that cumulative impairments in multiple domains affect functional reserve of swallowing with age, but the multifactorial etiology and postulated compensatory strategies of the brain are incompletely understood. This study investigates presbyphagia and its neural correlates, focusing on the clinical determinants associated with adaptive neuroplasticity.

**Materials and methods:**

64 subjects over 70 years of age free of typical diseases explaining dysphagia received comprehensive workup including flexible endoscopic evaluation of swallowing (FEES), magnetoencephalography (MEG) during swallowing and pharyngeal stimulation, volumetry of swallowing muscles, laboratory analyzes, and assessment of hand-grip-strength, nutritional status, frailty, olfaction, cognition and mental health. Neural MEG activation was compared between participants with and without presbyphagia in FEES, and associated clinical influencing factors were analyzed. Presbyphagia was defined as the presence of oropharyngeal swallowing alterations e.g., penetration, aspiration, pharyngeal residue pooling or premature bolus spillage into the piriform sinus and/or laryngeal vestibule.

**Results:**

32 of 64 participants showed swallowing alterations, mainly characterized by pharyngeal residue, whereas the airway was rarely compromised. In the MEG analysis, participants with presbyphagia activated an increased cortical sensorimotor network during swallowing. As major clinical determinant, participants with swallowing alterations exhibited reduced pharyngeal sensation. Presbyphagia was an independent predictor of a reduced nutritional status in a linear regression model.

**Conclusions:**

Swallowing alterations frequently occur in otherwise healthy older adults and are associated with decreased nutritional status. Increased sensorimotor cortical activation may constitute a compensation attempt to uphold swallowing function due to sensory decline. Further studies are needed to clarify whether the swallowing alterations observed can be considered physiological *per se* or whether the concept of presbyphagia may need to be extended to a theory with a continuous transition between presbyphagia and dysphagia.

## Introduction

Oropharyngeal dysphagia (OD) frequently occurs in various geriatric patient groups, such as acute hospitalized geriatric patients (Cabré et al., 2014), nursing homes residents (Lin et al., 2002) but also community dwelling older adults (Roy et al., 2007; Holland et al., 2011; Serra-Prat et al., 2011; Yang et al., 2013; González-Fernández et al., 2014; Madhavan et al., 2016). Swallowing disorders not only impair the quality of life, but further lead to severe complications such as malnutrition and aspiration pneumonia, thus increasing mortality (Cabre et al., 2010; Serra-Prat et al., 2012). A number of age-related diseases such as stroke (Yang et al., 2013; Labeit et al., 2018; Warnecke et al., 2021b), Parkinson’s disease (Kalf et al., 2012; Warnecke et al., 2021b), and neuromuscular disorders (Labeit et al., 2020b, 2021b; Warnecke et al., 2021a,b), play a decisive role as predisposing factors. Much less research has been devoted to age-related changes of oropharyngeal swallowing in otherwise healthy elderly individuals, also referred to as presbyphagia (Azzolino et al., 2019; Namasivayam-MacDonald and Riquelme, 2019).

The study data on the characteristics and frequency of swallowing alterations in healthy older adults is inconclusive, e.g., some studies report frequent penetration, aspiration or pharyngeal bolus residue (Butler et al., 2009a,b, 2010; Humbert et al., 2009; Omari et al., 2014; Jardine et al., 2020), other studies suggest the opposite (Mulheren et al., 2018; Garand et al., 2019; Jardine et al., 2020). Within this continuum, it is particularly difficult to decide whether such changes should be considered physiological or whether they represent a dysfunction or disease (Ortega et al., 2017; de Lima Alvarenga et al., 2018). A common model in this context is that age-related changes alone (primary presbyphagia) are not considered pathological. However, with the onset of additional disease, presbyphagia may limit compensatory reserve, resulting in manifest dysphagia due to the cumulative effect of disease and age-related changes in oropharyngeal swallowing. The component of age-related changes in the presence of additional disease with resulting dysphagia is then referred to as secondary presbyphagia (Warnecke et al., 2019).

Pathophysiological factors that are assumed to contribute to presbyphagia include sarcopenia (Zhao et al., 2018; Azzolino et al., 2019), pharyngeal hypesthesia (Shaker et al., 1994; Aviv, 1997; Labeit et al., 2019; Tomsen et al., 2021), decreased levels of substance P (Tomsen et al., 2021) which is released from pharyngeal sensory nerve endings into saliva, and anatomical changes, e.g., deterioration of the dentition and skeletal system (Cichero, 2018; Yin et al., 2018). Further, the central control of swallowing has come into focus in recent years. Clinical data and neuroimaging research has drawn attention to a widely distributed cortical and subcortical brain network (Malandraki et al., 2011a; Labeit et al., 2020a). Study results in Parkinson’s disease and stroke suggest adaptive cortical neuroplasticity and allocation of attentional resources as a compensatory mechanism for the disease-related swallowing dysfunction (Teismann et al., 2011; Suntrup et al., 2013a; Labeit et al., 2020a). In addition, neurostimulation techniques in dysphagia rehabilitation also modulate the cortical swallowing network (Suntrup et al., 2013b,^2015^). However, there are only few studies investigating age-related changes in central swallowing control (Martin et al., 2007; Humbert et al., 2009; Malandraki et al., 2010, 2011b; Teismann et al., 2010; Windel et al., 2015; Tae et al., 2021), with partly inconclusive results: Some studies indicate an increase in cortical activation (Martin et al., 2007; Humbert et al., 2009; Teismann et al., 2010), while other studies assume that the swallowing network remains essentially stable regardless of age (Windel et al., 2015) or even decreases in somatosensory areas (Malandraki et al., 2011b; Tae et al., 2021). Common to all of these studies is that neither swallowing function itself nor clinical parameters were related to neuroimaging findings. Moreover, group sizes were mostly small, suggesting underpowering of trials.

To comprehensively investigate presbyphagia and its neural correlates, the objectives of this study were (1) to determine the prevalence and pattern of age-related oropharyngeal swallowing alterations in individuals without neurological or ENT-diseases associated with dysphagia, (2) to investigate the underlying neural swallowing network changes in older adults with vs. without presbyphagia, (3) to determine the clinical influencing factors associated with adaptive neuroplasticity in aging, and (4) to assess the associated consequences of presbyphagia. For this purpose, the participants were characterized applying objective dysphagia diagnostics, magnetoencephalography (MEG) as a neuroimaging technique and a multidimensional geriatric assessment.

## Materials and methods

### Participants

64 people ≥ 70 years of age were recruited for this prospective cross-sectional study. Recruitment took place through newspaper and flyer advertisements or presentations at senior citizens’ meetings in the vicinity. Subjects were only included if there was no previously diagnosed neurological or structural disease associated with oropharyngeal dysphagia (stroke, Parkinson’s disease, dementia, neuromuscular disorder, neuroinflammatory disorder of the central nervous system, oropharynx carcinoma, laryngeal carcinoma, other carcinoma of the esophagus or upper gastrointestinal tract, condition after irradiation in the head and neck region). In addition, the subjects had to be community dwelling. Furthermore, there were no exclusion criteria based on limited mobility or limitations in activities of daily living. Participation in the study was possible regardless of the presence of a subjective or pre-diagnosed swallowing dysfunction.

### Multidimensional clinical and geriatric assessment

#### Demographic and clinical assessment:

The subjects age, gender, weight, height and hospitalization due to pneumonia within the last year were noted.

#### Swallowing assessment:

All participants underwent flexible endoscopic evaluation of swallowing (FEES), which, along with videofluoroscopy, is considered the diagnostic gold standard for dysphagia (Labeit et al., 2021a). FEES was performed following a stepwise protocol with testing of 3 different consistencies in the following order: three trials of 8 ml of green jelly (semisolid), three trials of 5 ml blue-dyed liquid, and 3 trials of white bread (solid) with a size of approximately 3 cm × 3 cm × 0.5 cm. The following swallowing dimensions were assessed for each swallowing trial: (1) premature bolus spillage, (2) penetration and aspiration, (3) residue in the pharynx. Depending on these swallowing dimensions, the presence of swallowing alterations were determined as follows: (1) no swallowing alterations. (2) swallowing alterations without airway compromise: premature bolus spillage at least into the piriform sinus in at least 2 out of 3 swallows of at least 1 consistency or/and pharyngeal residue pooling > coating according to the following scale (Warnecke et al., 2016) in at least 2 out of 3 swallows (determined after spontaneous clearing swallows if present) of at least 1 consistency but no penetration or aspiration. (3) swallowing alterations with airway compromise: presence of penetration and/or aspiration. In case of swallowing alterations, these were classified according to a previously published phenomenological FEES-classification (Warnecke et al., 2021b). To further quantify global swallowing function, a previously established swallowing-score was applied, rating each of the swallowing pathologies (premature bolus spillage, penetration/aspiration and residue in the pharynx) on a scale from 0 (normal) to 4 (severe impairment) for every trial and each food consistency, contributing to an overall cumulative score ranging from 0 to 108 (Warnecke et al., 2016). FEES rating was performed by a neurologist with more than 3 years of experience in the field of neurogenic dysphagia. The rating was blinded to the other clinical scores and parameters.

Participants were also asked to fill out the validated Swallowing Quality of Life Questionnaire “SwalQoL” to evaluate swallowing function-specific quality of life (McHorney et al., 2000).

#### Assessment of oropharyngeal sensory functions:

Subsequently, pharyngeal sensory testing was performed according to a previously validated FEES-based protocol. In brief, pharyngeal sensation was quantified by the latency of the swallowing response after pharyngeal topical application of 0.2, 0.3, 0.4, and 0.5 ml of liquid *via* a transnasally inserted eight Charrière infant feeding tube (Labeit et al., 2019).

In addition, smell and taste were tested with the validated 12-item Sniffin’ Sticks test (Burghart Medical Technology, Wedel, Germany). Norm databases have been established for age-standardized comparisons (Hummel et al., 2001). Four additional spray bottles were used to qualitatively assess the four basic qualities of taste (sweet, sour, salty, bitter) which were either recognized correctly or not.

To determine substance P level in saliva, which is released from pharyngeal sensory nerve endings and relates to swallowing function, e.g., in geriatric stroke patients and Parkinson’s disease, (Arai et al., 2003; Schröder et al., 2019) a saliva probe was taken from each participant using a salivette (Sarstedt, Nuembrecht, Germany). Probes were centrifuged immediately at 4000 rpm for five minutes. Supernatants were stored in a deep freezer at –20°C. Substance P level was determined by a commercially available competitive ELISA-type immunoassay according to the manufacturer’s instructions (SP Immnunoassay, catalog no. KGE007; R&D Systems, Minneapolis, MN, United States).

#### Assessment of nutritional status, muscle strength/volume and frailty:

To characterize body composition, a bioimpedance analysis was done (Kyle et al., 2003) using a phase sensitive multifrequency analyzer (model BIA 2000-M, Data Input GmbH, Frankfurt, Germany) with the according analysis software (Nutri Plus, also Data Input GmbH) recording fat free mass (FFM) index, fat mass (FM) index, extracellular mass to body cell mass (ECM/BCM) ratio, and phase angle. An increased ECM/BCM ratio was used as a surrogate marker for decreased nutritional status, as this parameter has been validated as a surrogate marker for malnutrition in various patient groups (Adami et al., 1993; Talluri et al., 1999; Avram et al., 2010; Pelzer et al., 2010; Małecka-Massalska et al., 2014; Staufer et al., 2018).

In addition, MRI muscle volumetry of the digastric and geniohyoid muscles was performed. MRI was performed using a 3 Tesla MRI scanner (Philips Ingenia 3.0T,Philips, Eindhoven, The Netherlands) to obtain coronal and axial T1-weighted images (TE/TR = 7508/595267 ms, TI = 0 ms, slice thickness = 4.4 mm) using a phased array coil. The geniohyoid and digastric muscles on both sides were segmented slice by slice using Medical Imaging Toolkit (MITK 2.4.0.0, Heidelberg, Germany). Segmentations were then adjusted semi-automatically in the other dimensions. Overall muscle volume of these muscles was determined. This procedure of muscle volumetry was previously validated and is described in detail elsewhere (Sporns et al., 2018). The Mini Nutritional Assessment – Short Form (MNA-SF) was additionally applied as a malnutrition screening tool (Rubenstein et al., 2001).

To evaluate skeletal muscle force, hand-grip-strength was measured in a standardized manner using a Jamar dynamometer on the dominant hand, in sitting position, with elbows bent at a 90° angle. Three measurements were taken at intervals of one minute, whereas the highest value was recorded in kilogram.

As a screening for frailty the 5-item simple frailty (FRAIL) questionnaire was applied (Morley et al., 2012).

#### Cognitive and psychological assessment:

A screening for cognitive impairment was performed using the Montreal Cognitive Assessment (MoCA) (Nasreddine et al., 2005). In addition, the ability to cope with everyday life was evaluated applying the Bayer Activities of Daily Living Scale (B-ADL) (Hindmarch et al., 1998). The 15-item short form of the Geriatric Depression Scale (GDS) was used to screen for affective symptoms potentially influencing eating desire (Yesavage and Sheikh, 1986).

### Magnetoencephalography (MEG) data acquisition and analysis

Magnetoencephalography is a non-invasive, functional neuroimaging procedure in which the magnetic activity of the brain is measured *via* external sensors. In this study, MEG was used to compare sensorimotor swallowing network activation in older adults with vs. without swallowing alterations as diagnosed by FEES. The MEG measurements were performed with a 275-channel whole-head device (Omega2005 WC, CTF Systems Inc., Canada). Magnetic fields were recorded with a sampling rate of 600 Hz and an online low pass filter of 150 Hz. A swallowing paradigm and a pharyngeal sensory stimulation paradigm were recorded to investigate motor and sensory aspects of oropharyngeal swallowing. Both paradigms were previously established and are described in detail elsewhere (Teismann et al., 2009; Suntrup et al., 2013b).

Magnetoencephalography data analysis followed a standard pipeline established by our group and applied in manifold previous studies on the cortical control of swallowing (Suntrup et al., 2013b,^2015^; Muhle et al., 2018). In summary, the MATLAB-based (MathWorks Inc., USA) software toolbox “FieldTrip”^1^ was used (Oostenveld et al., 2011). At first, MEG raw data was filtered within theta (4–8 Hz), alpha (8–13 Hz), beta (13–30 Hz), low gamma (30–60 Hz) and high gamma (60–80 Hz) frequency ranges. Swallowing acts were identified according to coregistered EMG data. For pneumatic stimulation, trials contaminated by swallowing were rejected. Source localization of each subject’s task-related cortical activation changes for both paradigms was done within each frequency range by means of a linearly constrained minimum variance (LCMV) beamformer technique. After that, three-dimensional source localization maps were spatially normalized to a template MNI brain (T1, Montreal Neurological Institute, Canada) using SPM8^2^ to compute grandaverages across subject groups. To identify significant (*p* < 0.05) brain activation differences between participants with vs. without swallowing alterations, data from both MEG paradigms were compared between groups applying a cluster-based non-parametric randomization approach built into FieldTrip.

## Statistical analysis

Descriptive statistics were used to describe prevalence and pattern of swallowing dysfunction in our study cohort. Participants were categorized into those with vs. without swallowing alterations according to FEES diagnosis and the average swallowing sum score was determined for each group. MEG results of these two groups were statistically compared as described above. To identify clinical influencing factors associated with the differences in brain activation found in MEG, the further assessed parameters for pharyngeal sensory function, reduced nutritional status, frailty, hand-grip-strength, volume of swallowing muscles, cognitive and affective function were compared between groups.

To assess associations with outcome parameters in older age, differences in pneumonia incidence, Swal-QoL, MNA-SF, bioimpedance parameters such as ECM/BCM ratio and Body Mass Index (BMI) were calculated.

For metric data, the independent sample-t-test (two-sided), for ordinal and non-parametric data the Mann-Whitney-U-test and for nominal data the chi-square-test was used. Further, a linear regression model was applied to assess associations with an elevated ECM/BCM ratio as a surrogate marker for a reduced nutritional status, using age, gender and swallowing-score as predictors.

## Results

### Demographic characteristics, prevalence and pattern of swallowing alterations

Of the 64 participants, 25 (39.1%) were men and 39 (60.9%) women. The average age was 77.7 ± 6.1 years. Half of the participants showed normal swallowing function (*N* = 32). Twenty-nine (45.3%) participants had swallowing alterations without airway compromise. Three (4.7%) subjects had more severe swallowing alterations with penetration and/or aspiration. Of the subjects with swallowing alterations, the majority (*N* = 25, 39.1%) had residue with focus in the valleculae as phenotype, five (7.8%) subjects had a complex phenotype in which both residue and premature spillage occurred equivalently, while 2 (3.1%) subjects had premature bolus spillage as leading phenotype.

### Magnetoencephalography (MEG) results

In three subjects no MEG could be recorded due to technical problems (*N* = 2) or participant refusal (N = 1), one further subject was excluded from MEG swallowing data analysis because EMG recording of swallowing acts had not worked out. In a further 10 participants, pharyngeal sensory stimulation could not be performed successfully in the MEG (technical issues = 6, refused or quitted measurement = 4).

Similar subject performance during the MEG measurement in groups with vs. without swallowing alterations was confirmed by similar swallow count (63 ± 19 vs. 64 ± 20, *p* = 0.854) and head movement (swallow paradigm: 7.0 ± 4.4 mm vs. 7.9 ± 9.9 mm, *p* = 0.621, pharyngeal sensory stimulation: 7.4 ± 3.9 mm vs. 7.6 ± 3.1 mm, *p* = 0.856). Both groups showed comparable EMG power of submental muscles within the swallow interval (88.1 ± 85.0 μV vs. 74.0 ± 48.1 μV, *p* = 0.430) and similar EMG peak-to-peak amplitude (382.2 ± 202.6 μV vs. 395.6 ± 227.5 μV, *p* = 0.810).

**FIGURE 1:**
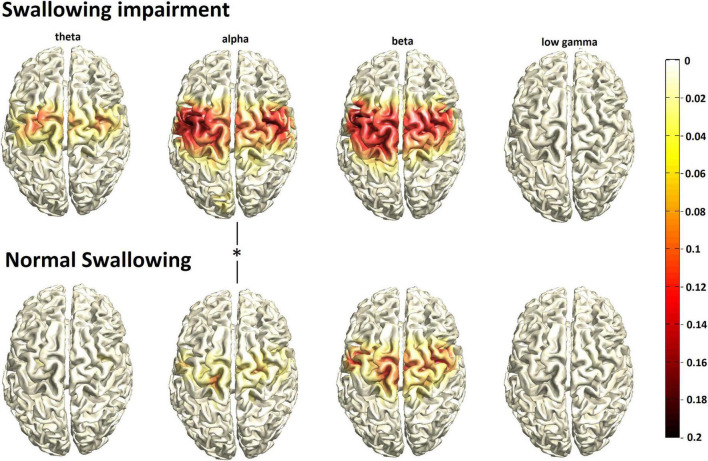
Group mean swallowing associated cortical activation (N = 30/group) in participants with swallowing alterations (top row) vs. normal swallowing function (bottom row). The color bar represents power changes relative to the resting state, the asterisk indicates statistical significant differences.

During swallowing, event-related desynchronization of oscillatory brain activity was found from the theta to the low gamma range in both groups without changes in high gamma. With normal swallowing, activation was more focal in space, involving mainly the bilateral pericentral cortex corresponding to primary sensorimotor areas with peak activation located in the swallowing primary motor area (peak value, [MNI coordinates], location): theta: –0.0726, [–29, –12,49], left precentral gyrus; alpha: –0.1258, [19, –14, 50], right precentral gyrus; beta:-0.1885, [46, –2,32], right precentral gyrus; low gamma: –0.0213, [28, –4,60], right precentral gyrus), but also more focal in frequency, centering in the “sensorimotor” alpha and beta frequency bands with little activation in adjacent theta and low gamma ranges (Figure 1, bottom row). Contrary to that, the group with swallowing alterations (Figure 1, upper row) displayed stronger and broader activation in space with additional recruitment of secondary sensorimotor regions (peak value, [MNI coordinates], location: theta: –0.1180, [–28, –4,61], left precentral gyrus; alpha: –0.1931, [38, –12,40], right precentral gyrus; beta: –0.2064, [39, –2,32], right precentral gyrus; low gamma: –0.0162, [–28, –4,61], left precentral gyrus). Moreover, activation was found in a broader frequency range with additional prominent theta activation. Statistical comparison revealed significantly stronger event-related desynchronization in the alpha range in the participants with swallowing alterations (***p*** = 0.024).

**FIGURE 2:**
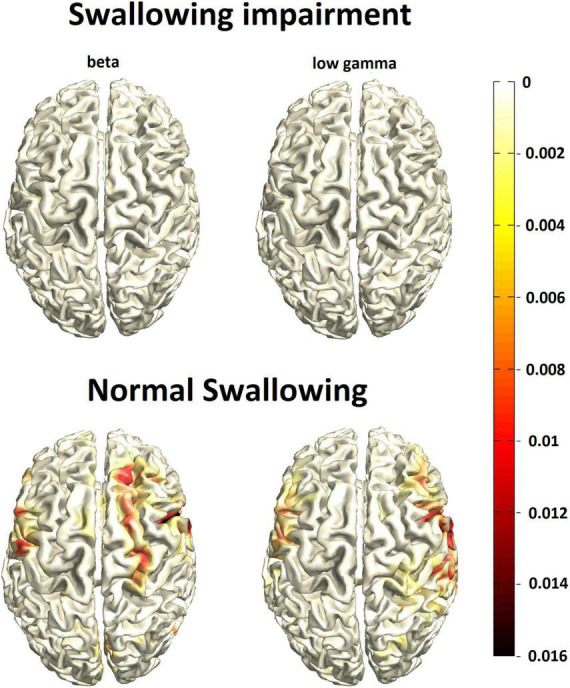
Group mean cortical activation response to pharyngeal sensory stimulation (N = 27/group) in participants with swallowing alterations (top row) vs. normal swallowing function (bottom row). The color bar represents power changes relative to the resting state.

As expected, event-related desynchronization evoked by pharyngeal sensory stimulation was weaker compared to swallowing in both groups and found more lateral in the pericentral cortex in the beta and low gamma range (Figure 2). Opposite to the activation pattern observed with the motor task of swallowing, pure sensory stimulation resulted in relatively stronger event-related desynchronization in normal swallowers compared to participants with swallowing alterations. However, due to the weakness of the MEG signal with a worse signal-to-noise ratio, this difference missed statistical significance.

### Biological and clinical determinants of cortical activation changes

Demographic characteristics and results of the multidimensional clinical and geriatric assessment are displayed in Table 1 (with additional graphical visualization of the data in Supplementary material 1). Regarding potential clinical determinants for the observed cortical activation changes seen with MEG, pharyngeal sensory impairment was the major factor in those participants with presbyphagia. Both groups differed significantly in the swallowing latency results at 0.2, 0.3, and 0.4ml. Results indicate pharyngeal hypesthesia with resulting delayed swallowing response in subjects with presbyphagia (matching the validation study of this test procedure in which pharyngeal topical anesthesia led to increased swallowing latency with the largest effect size at 0.4 ml [Labeit et al., 2019)]. Depression as assessed with the GDS was at least close to significance (*p* = 0.056) but scores were generally low (norm = 0–5 pts). All other parameters showed no significant differences between the groups.

**TABLE 1:** Clinical comparision of the participants with vs. without swallowing alterations.

Domain	Swallowing alterations (*n* = 30)	Normal swallowing (*n* = 30)	*P*-value
Age, years, mean ± SD	78.6 ± 5.9	75.8 ± 5.7	0.067
Men, n (%)	14 (47%)	10 (33%)	0.292
Swallowing score, mean ± SD	18.8 ± 12.4	8.7 ± 4.3	<0.001*
Subscore residue semisolid, median (range)	1 (0–11)	0 (0–4)	0.009*
Subscore residue liquid, median (range)	0 (0–12)	0 (0–3)	0.147
Subscore residue solid, median (range)	7 (1–10)	2 (0–4)	<0.001*
**Pharyngeal sensory functions:**			
Swallowing latency with 0.2 ml, s, mean ± SD	2.3 ± 0.8	1.8 ± 0.7	0.017*
Swallowing latency with 0.3 ml, s, mean ± SD	2.2 ± 0.7	1.6 ± 0.5	0.001*
Swallowing latency with 0.4 ml, s, mean ± SD	1.9 ± 0.7	1.2 ± 0.6	0.001*
Swallowing latency with 0.5 ml, s, mean ± SD	1.6 ± 0.7	1.3 ± 0.6	0.072
Substance P, pg/ml, mean ± SD	53.6 ± 25.0	61.3 ± 67.7	0.559
Sniffin’-sticks score, median (range)	9 (2–12)	9 (4–11)	0.267
Taste score, median (range)	4 (2–4)	4 (2–4)	0.934
**Muscle assessment**			
Hand grip strength, kg, mean ± SD	26.1 ± 8.5	28.0 ± 9.4	0.404
Swallowing muscle volume, mm^3^, mean ± SD	7263 ± 2444	6929 ± 2330	0.631
**Cognition and mental health:**			
MoCA, median (range)	25 (18–30)	25 (20–29)	0.622
B-ADL, median ± SD	1.6 ± 0.7	1.6 ± 0.5	0.265
GDS, median (range)	2 (0–7)	1 (0–6)	0.056
**Outcomes:**			
Swal QoL, median (range)	200 (131–220)	211 (135–220)	0.067
FFM-Index kg/m^2^, mean ± SD	19.0 ± 2.9	18.6 ± 2.2	0.569
FM-Index kg/m^2^, mean ± SD	7.8 ± 3.5	8.0 ± 2.7	0.814
ECM/BCM ratio, mean ± SD	1.4 ± 0.4	1.3 ± 0.2	0.087
Phase angle, mean ± SD	4.4 ± 0.9	4.7 ± 0.5	0.163
BMI, kg/m^2^, mean ± SD	26.9 ± 4.7	26.6 ± 3.7	0.804
MNA-SF, median (range)	13 (4–14)	13 (7–14)	0.795
FRAIL, median (range)	1 (0–4)	0 (0–3)	0.030*
Previous hospitalization due to pneumonia, n (%)	2 (6.7%)	0 (0)	0.492

Clinical comparison of the participants with vs. without swallowing alterations: B-ADL, Bayer- Activities of Daily Living; BMI, body mass index; FRAIL, simple frailty-questionnaire; GDS, Geriatric Depression Scale; MNA-SF, Mini Nutritional Assessment – Short Form; MoCA, Montreal Cognitive Assessment; SwalQoL, Swallowing Quality of life questionnaire; FFM, fat free mass; FM, fat mass; ECM/BCM, extracellular mass to body cell mass. Bold values are significant *p*-values.

Outcome of swallowing dysfunction: Two participants had been hospitalized for pneumonia within the last year. Both of them showed dysphagia with airway compromise in FEES. Swallowing associated quality of life (max. achievable SwalQol-Score: 220) tended to be lower in subjects with swallowing alterations but the difference was not statistically significant in our community-dwelling population (Table 1). The FRAIL score differed significantly indicating more cases with prefrailty (FRAIL scores 1-2) in participants with swallowing alterations (Table 1). All other parameters showed no significant differences between the groups.

When analyzing the whole study population as a single group, a significant multiple linear regression equation was found to predict an increased ECM/BCM ratio as indicator of a reduced nutritional status [F(3,60) = 26.687, *p* = 4.3E–11 with R-squared of 0.57]. Older age, female gender, and worse swallowing function score were all independent predictors for an increased ECM/BCM ratio indicative of reduced nutritional status (Table 2).

**TABLE 2:** Predictors of reduced nutritional status.

Predictor	Regression coefficient [95% CI]	*P*-value
Age	0.015 [0.05–0.025]	0.003*
Female gender	0.243 [0.127–0.359]	1.1E–4*
Swallowing score	0.019 [0.013–0.025]	5.0E–9*

Results of the multiple linear regression model to predict an increased extracellular mass to body cell mass ratio (ECM/BCM ratio). Bold values are significant *p*-values.

## Discussion

### Prevalence and pattern of presbyphagia

Our results show that swallowing alterations are common in healthy older people, being present in half of the participants. Mostly, presbyphagia presented without airway compromise, e.g., pharyngeal residue in the valleculae, more rarely premature bolus spillage or a combination of both. Nevertheless, in individual cases (5% of participants) penetration or aspiration also occurred. These results are partly consistent with a systematic review on swallowing changes in healthy people over 85 years of age. Here too, only few studies detected airway compromise with aspiration, however, penetration was more frequently reported compared to our cohort. Pharyngeal residue, which was the most common swallowing alteration in our study, was reported with varying prevalence rates from none or trace to nearly 70%, but only few studies commented on residue at all (Jardine et al., 2020). As observed in our study, prevalence of pharyngeal hypesthesia increases with age (Aviv, 1997) and is related to delayed pharyngeal swallowing reaction (Shaker et al., 1994) and reduced perception of pharyngeal residue that are subsequently not cleared. There are contradictory study results for penetration and aspiration: Some authors consider them to be rare (Mulheren et al., 2018; Garand et al., 2019) and not age-related (Garand et al., 2019), while others report frequent airway compromise in old age (Butler et al., 2009a,b, 2010). A possible explanation could be that both findings are not a frequent symptom of primary presbyphagia itself, but rather result from (age-related) diseases such as stroke and Parkinson’s disease. These conditions may have been underdetected to varying degrees in the reported cohorts. In addition, differences in study design such as different diagnostic procedures may have played a role. On the other hand, age-related swallowing deterioration may be seen as a continuum from insignificant swallowing alterations to manifest dysphagia with airway compromise (de Lima Alvarenga et al., 2018).

### Biological determinants of presbyphagia

Pharyngeal hypesthesia as indicated by increased swallowing response latency in sensory testing was the only significant difference from the group with regular swallowing function. Consistent with our results, increased sensory discrimination thresholds in the mouth and pharynx have been observed in older adults (Aviv, 1997). It has also been shown that older adults need larger bolus volumes, i.e., a stronger sensory stimulus, to sufficiently trigger the swallow response (Shaker et al., 1994). The underlying mechanisms of this sensory decline are not well understood. Degeneration of substance P-releasing sensory nerve endings is suggested, as oropharyngeal dysphagia in older adults was associated with pharyngeal hypesthesia and decreased substance P-levels in saliva (Tomsen et al., 2021). Further, there is growing evidence in geriatric patients and nursing home residents for a beneficial effect of substance P-releasing drugs, e.g., capsaicin on swallowing function (Ebihara et al., 2005; Rofes et al., 2013). However, there was no singificant difference in substance P-level between the subjects with presbyphagia and normal swallowing function in this study.

Besides sensory deficits, there were no other identifiable contributors to presbyphagia such as reduced volume of swallowing muscles or cognitive impairment in our community-dwelling older adults. However, following the concept of reduced functional reserve, in case of an additional acute disease causing dysphagia, e.g., stroke, previous studies have found that preexisting reduced volume of swallowing muscles and cognitive function influence the severity of dysphagia (Jo et al., 2017; Sporns et al., 2017). In other recent studies, it is not primarily a reduction in muscle volume but rather fat remodeling in both swallowing (Nakao et al., 2021a,b) and limb (Akazawa et al., 2019) muscles that is linked to dysphagia in older people/patients. Thus, in addition to the muscle volume, the quality or composition of the muscles could also play a relevant role. Interestingly, there was also no significant difference in age, which is consistent with a questionnaire study in community dwelling elderly (Roy et al., 2007). Therefore, similar to other geriatric syndromes, a distinction between chronological and biological age may be more appropriate (Williams et al., 2020). Nevertheless, when considering the entire age spectrum, the changes observed are most likely age-related alterations: They occur in young, healthy subjects much less frequently (Butler et al., 2009a) which is also evident from the fact that the swallowing score used in this study is significantly lower in young, healthy cohorts (Labeit et al., 2019).

### Magnetoencephalography (MEG) activation

Participants with swallowing alterations exhibited increased sensorimotor activation (event-related desynchronization) in the spatial and frequency domain in the central control of swallowing. Activation increase was especially observed in the alpha frequency range, which has specifically been linked to somatosensory processing, and was also prominent in the theta range. Theta oscillations have been related to self-directed movement (Kaplan et al., 2012) and are known to increase with task complexity (Caplan et al., 2001). Combined with the clinical data collected in our study – observed changes in presbyphagic elderly may constitute a compensation attempt to uphold swallowing function in the presence of a decline in oropharyngeal sensory function. However, the directionality of the relationship between neural correlation and swallowing cannot be established with certainty. Therefore it may also be possible that the neural activation changes in the presbyphagia group caused the swallowing alterations. However, the fact that the extent of event-related desynchronization during a motor task is related to task complexity and skill, reflecting energetic processes such as allocation of planning resources, arousal and effort (Studer et al., 2010) speaks against the latter hypothesis.

The principal sensorimotor activation pattern during swallowing observed here is congruent with previous MEG studies (Teismann et al., 2010; Suntrup et al., 2013b; Muhle et al., 2020). However, so far there are few studies that investigate neural correlates of age-related changes in swallowing and barely any study correlated objectively assessed swallowing function with neuroimaging results among older adults. The largest fMRI study by Windel et al. comparing the neural representation of swallowing in 27 healthy older volunteers (mean age 64.8 years, range 55–75 years) vs. a young group (mean age: 24 years) consistently revealed activation focusing in sensorimotor areas. Depending on the statistical analysis chosen, there was either no significant difference between the older and younger subjects, or a bilaterally increased activation in the Brodmann area 10 (= prefrontal cortex) in the older subjects. However, as a limiting factor, the relatively young age range and the absence of information on swallowing function must be taken into account. The authors conclude that the sensorimotor swallowing network essentially retains its functionality in old age but older adults utilized additional attention to uphold sufficient swallowing performance (Windel et al., 2015). In support of the hypothesis of a predominantly stable swallowing network across age, brain activation in our older adults with regular swallowing function was also close to the results of healthy young volunteers in a previous MEG study applying a similar swallowing paradigm (Teismann et al., 2010), i.e., displaying more focal activation in a narrower frequency range. This refutes the previously made assumption, that increased somatosensory processing may be a general pattern with aging indicating reduced processing efficiency or limited capacity for targeted inhibition irrespective of swallowing function (Teismann et al., 2010). Adding to the hypothesis of additional recruitment of brain resources, we also observed an activation increase in secondary sensory and motor areas in presbyphagic participants. Similarly, another fMRI study with 11 older subjects showed increased cortical activation including the pericentral and inferior frontal cortex compared to young control subjects. The authors suggest an increased effort related processing and give further examples of motor tasks that are associated with increased effort and cortical processing in old age (Humbert et al., 2009). In accordance to that, a significantly stronger activation was observed in older subjects with more demanding water bolus swallowing compared to simple saliva swallowing in the premotor and prefrontal cortex in a further study (Martin et al., 2007). Compensatory over-activation was attributed to reduced oral bolus control by the investigators, which may again have been caused by reduced sensation.

Contrary, in a methodologically different fMRI study on 46 healthy individuals (age range 19 - 73 years), the swallowing-task related activation in the precentral gyri, postcentral gyri, and insular cortices was inversely correlated with age and thus decreased in the older adults. However, age was associated with increased brain-activation in the default-mode-network, which is assumed to represent resting-state activation. The authors conclude that aging decreases task-related activation of the swallowing network and reduces task-induced deactivation of the default mode network (Tae et al., 2021). A further fMRI study on 9 healthy older participants (age range 66–77 years) showed reduced activation, especially in somatosensory areas compared to a young control group. The authors suggest, that the primary motor areas are preserved across age, whereas the somatosensory and sensorimotor integration declines (Malandraki et al., 2011b). Corroborating, we were now able to demonstrate that indeed reduced pharyngeal sensation seems to constitute the major determinant of primary presbyphagia and the observed neural activation changes. However, we cannot definitely say whether a decline in peripheral sensory nerve function with consecutively disturbed afferent feedback to the cortical swallowing network, or reduced capacity and efficacy in central sensory processing itself is the origin of sensory decline. Taking into account the compensatory additional allocation of secondary sensorimotor areas observed in our and previous imaging studies combined with less cortical response to pharyngeal sensory stimulation in our presbyphagic participants, one may favor the hypothesis of predominant decline in peripheral nerve function. The absence of substance P reduction, however, speaks against that.

Besides swallowing, the phenomenon of an age-related compensatory increase in primary and secondary motor areas has also been described for other motor tasks (Loibl et al., 2011). However, it is unclear whether such increased cortical recruitment represents an effective compensation mechanism. In analogy to studies on motor skill acquisition by training, less task-related activation reflects a reduction of cortical processing demands and/or increased processing efficiency (Studer et al., 2010). It has been shown that subjects who recruit more cerebral activation for simple motor tasks already show poorer motor performance with increasing task complexity (Loibl et al., 2011). Our subjects demonstrated swallowing alterations despite the observed activation increases in cortical processing, suggesting insufficient compensation. On the other hand, presbyphagia was mild in most of them and sufficient oral nutrition was possible.

### Consequences of swallowing alterations

Although our community-dwelling population was quite homogeneous with respect to outcome parameters irrespective of swallowing status, we observed more cases with prefrailty among presbyphagic elderly. Corroborating this, the degree of swallowing alteration was a highly significant predictor for an increased ECM/BCM ratio and thus for a reduced nutritional status. This underlines the medical relevance of presbyphagia. Further independent risk factors for a reduced nutritional status were increased age and female gender. These results are consistent with a population-based longitudinal cohort study in independently living older adults in which initial signs of oropharyngeal dysphagia were associated with malnutrition and pneumonia in a follow-up one year later (Serra-Prat et al., 2012). A Finnish study among residents of nursing homes also found a strong association between swallowing difficulties and malnutrition (Suominen et al., 2005). According to our data, this relationship holds true for the earliest stages of presbyphagia already. However, it should be noted that reduced nutritional status may not only be a consequence but also a cause of the swallowing alterations. Due to the cross-sectional design of this study, an assessment of the directionality of this observed association is not possible. Furthermore, an increased ECM/BCM ratio is only an early sign of a reduced nutritional status and not of malnutrition according to the usual diagnostic criteria. This is also reflected in the fact that there is no difference between subjects with and without swallowing alterations in the MNA, a commonly used screening parameter for the assessment of malnutrition. Since there were only two cases of pneumonia, no statistical analysis was appropriate. Nevertheless, both pneumonia subjects showed airway compromise, demonstrating that besides malnutrition and frailty, development of pneumonia may be another consequence in older adults with swallowing alterations.

### Limitations of the study

There are several limitations that must be taken into account when interpreting the results of this study. Most participants have responded to the study advertisement on their own initiative, which may have led to a selection bias. Despite taking the detailed patient history, it cannot be excluded that some participants suffered from undiagnosed neurological diseases associated with dysphagia. Neither skeletal system nor dental status were systematically assessed in the study, although both parameters are considered important factors in presbyphagia. The sample size chosen *a priori* was based on numbers typically required for group comparison in this type of MEG analysis. There may be underpowering for single clinical determinants studied, so there is a risk that relevant differences were not detected due to a beta error. Further, MEG can primarily assess cortical activity and is limited in the detection of subcortical brain function.

## Conclusion

Swallowing alterations i.e., pharyngeal residue are frequent in healthy elderly people and are associated with reduced pharyngeal sensation. Increased sensorimotor cortical activation may constitute a compensation attempt to uphold swallowing function in the presence of sensory decline. Swallowing alterations are associated with prefrailty and deterioration of the nutritional status. Future studies are therefore needed to investigate if the original concept of presbyphagia, in which age-related swallowing alterations are considered physiological *per se*, may need to be expanded. There may be a continuum between compensated presbyphagia and decompensated dysphagia, with the latter being associated with poorer outcome.

## Data availability statement

The data supporting the conclusions of this article can be found in the Supplementary Material.

## Ethics statement

The studies involving human participants were reviewed and approved by Ethik-Kommission der Ärztekammer Westfalen-Lippe und der Westfälischen Wilhelms-Universität Münster. The patients/participants provided their written informed consent to participate in this study.

## Author contributions

BL: study design, data collection, data analysis, and writing of the manuscript. RG, ThR, and PS: data analysis. PM, AW, ToR, AH-K, JG, and RW: data analysis and editing of the manuscript. JVI: data collection and data analysis. JS: data collection. IC: editing of the manuscript. TW and RD: conceptual design, editing of the manuscript. SS-K: conceptual design, data analysis, writing of the manuscript. All authors contributed to the article and approved the submitted version.

## Footnotes

1
http://www.ru.nl/fcdonders/fieldtrip

2
http://www.fil.ion.ucl.ac.uk/spm
