# Supplementary figures and images for: Clinical determinants and neural correlates of presbyphagia in community-dwelling older adults
Source: Front Aging Neurosci. 2022 Jul 28;14:912691. doi: 10.3389/fnagi.2022.912691 (PMC9366332; doi:10.3389/fnagi.2022.912691)

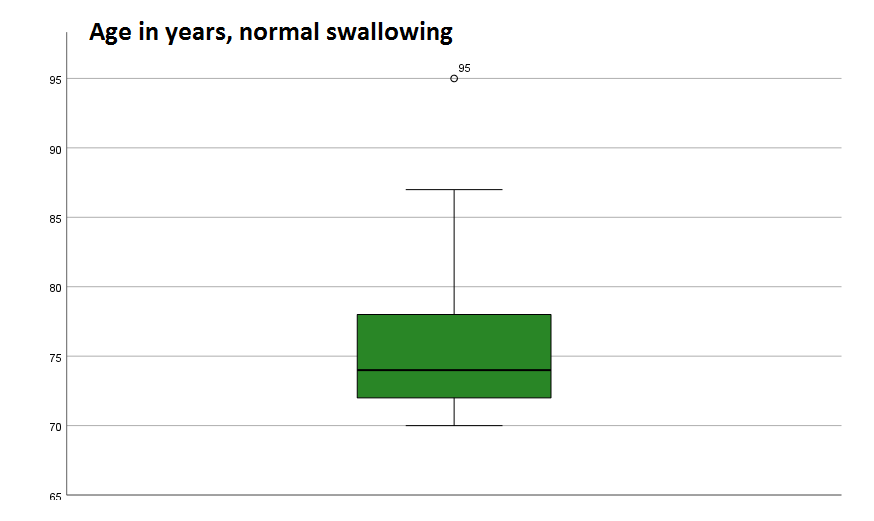


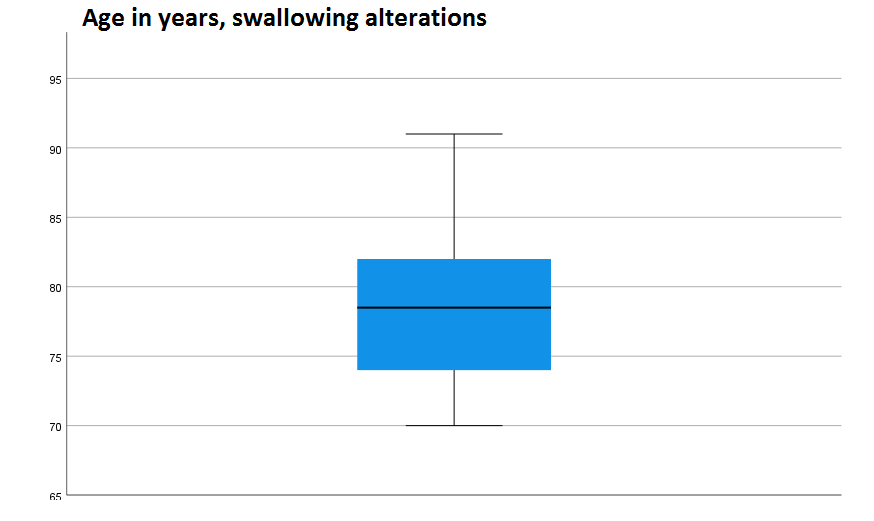


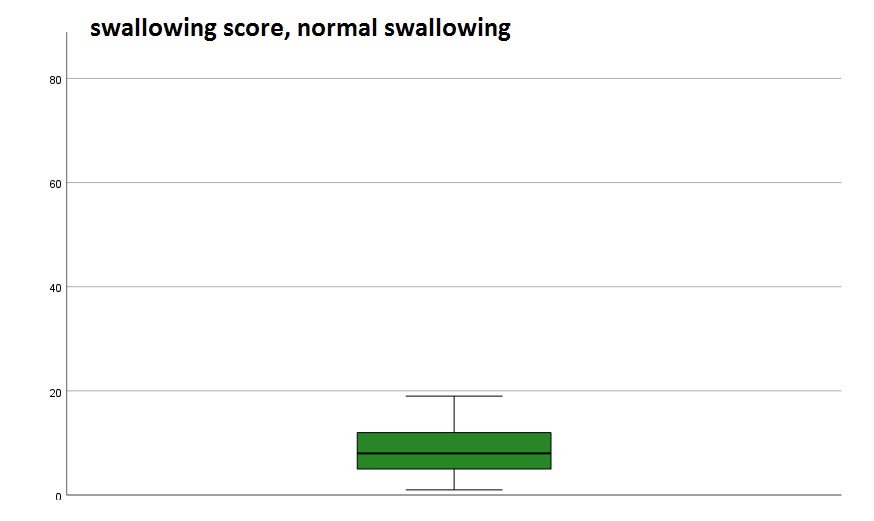


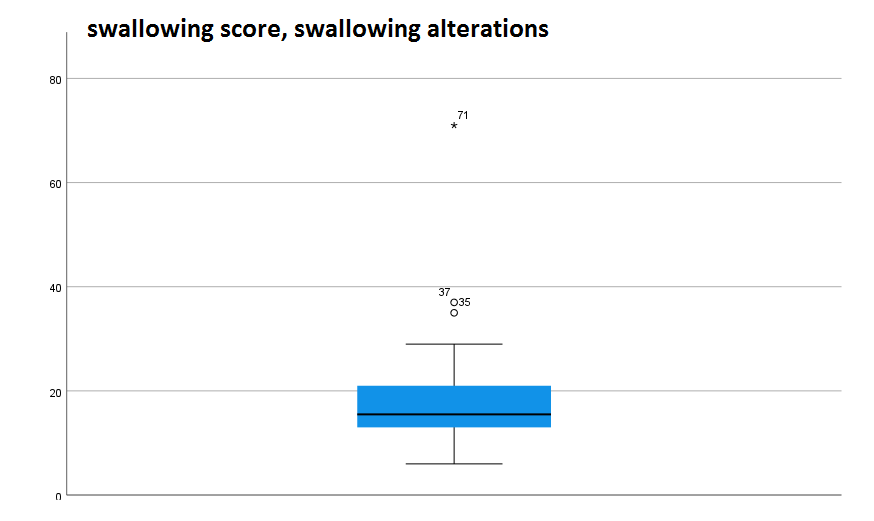


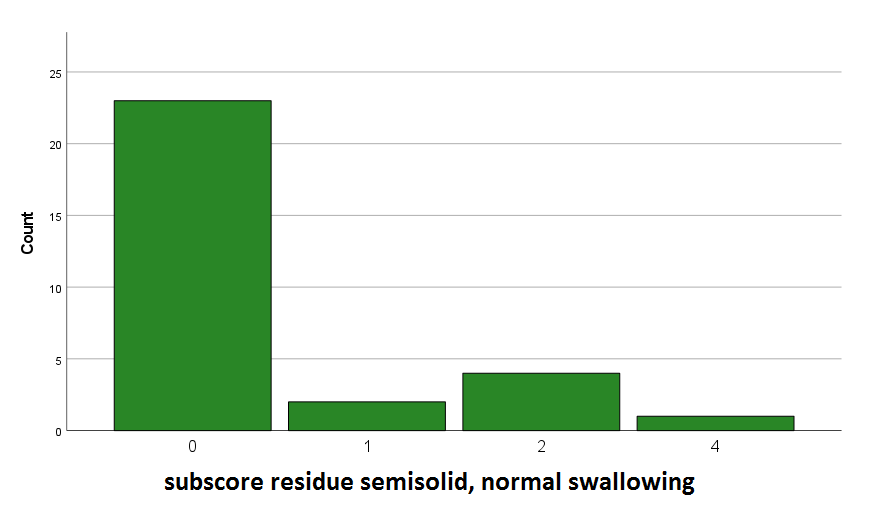


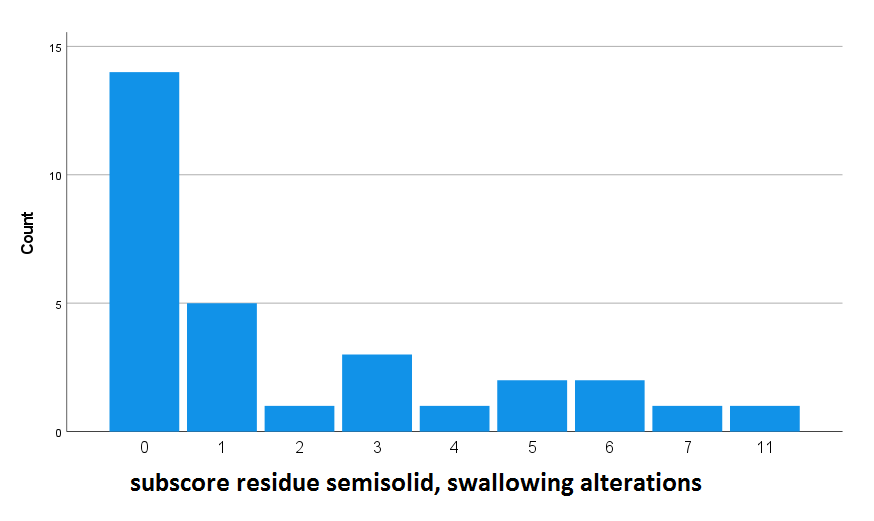


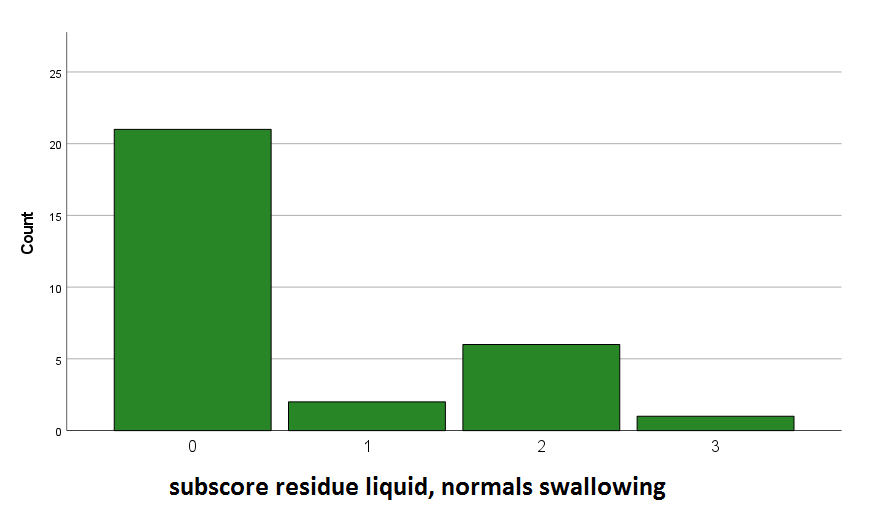


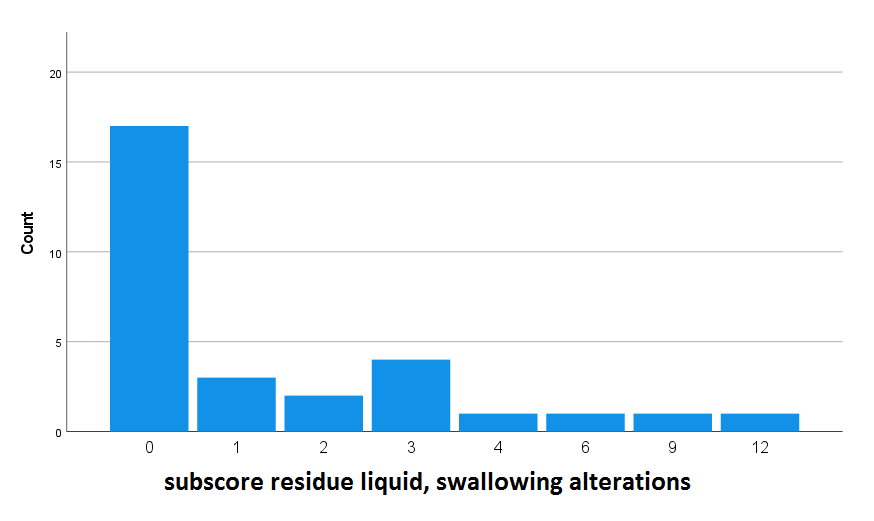


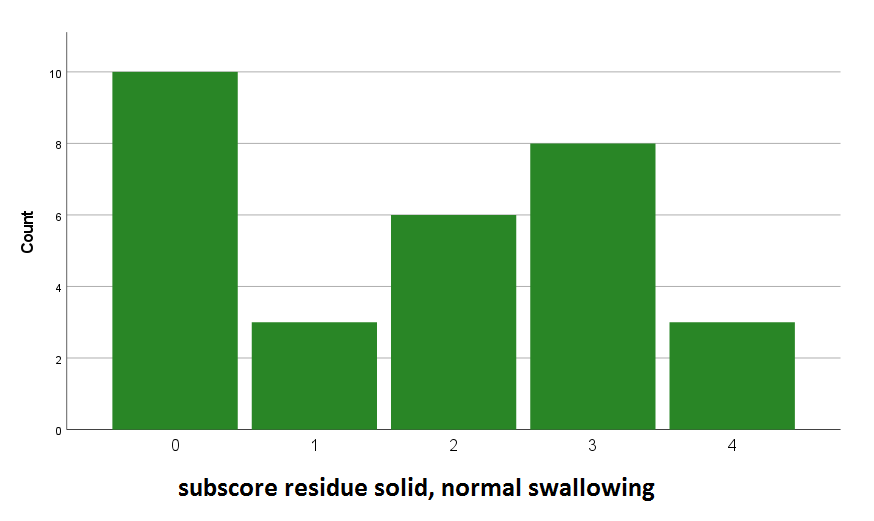


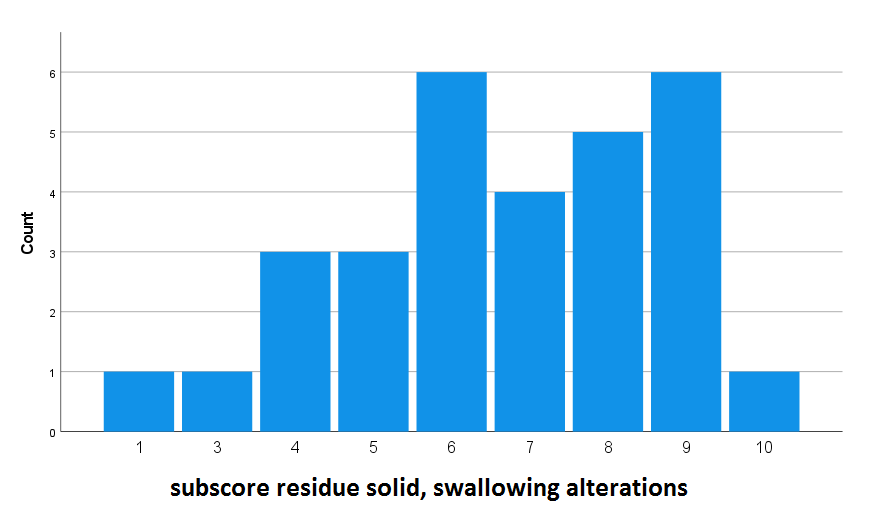


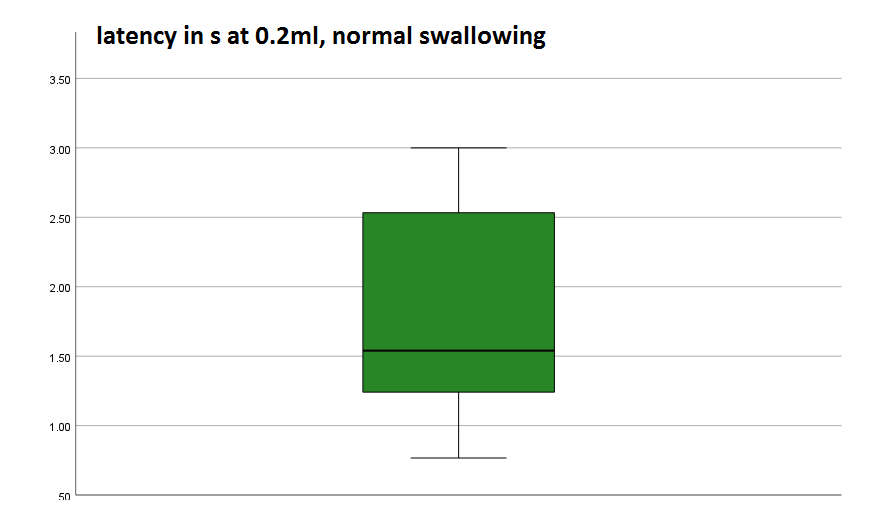


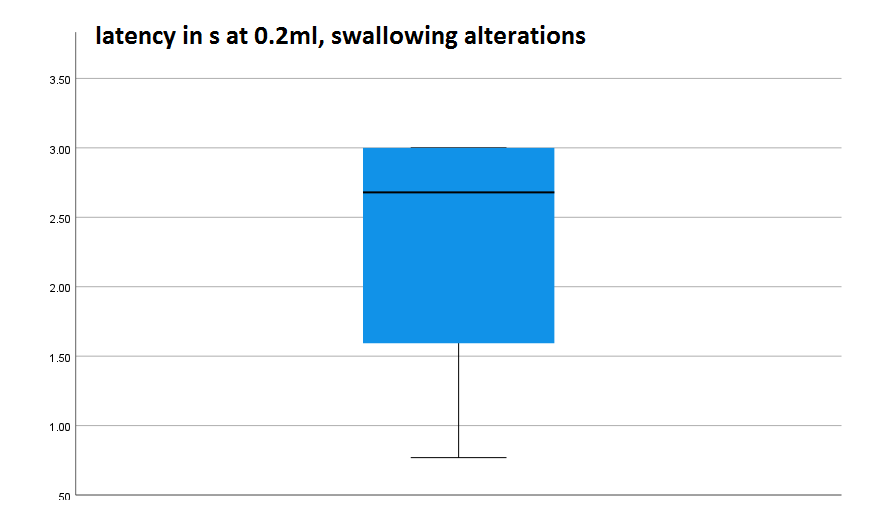


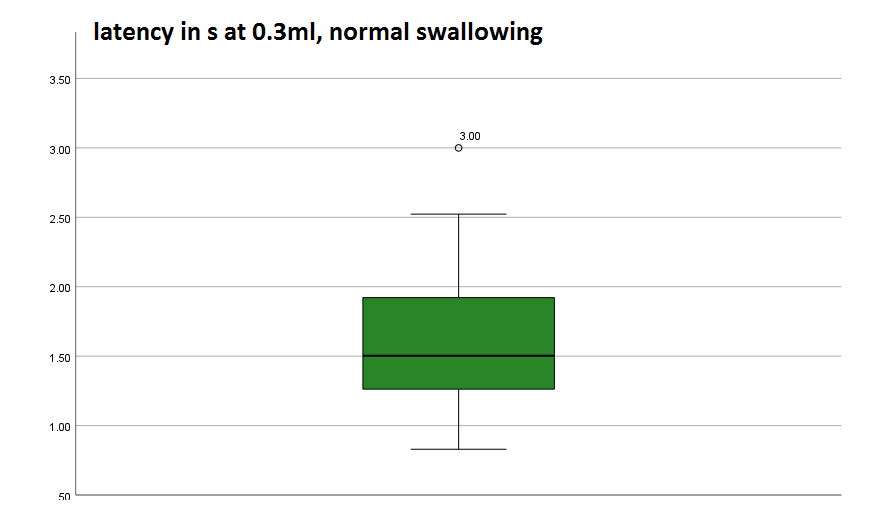


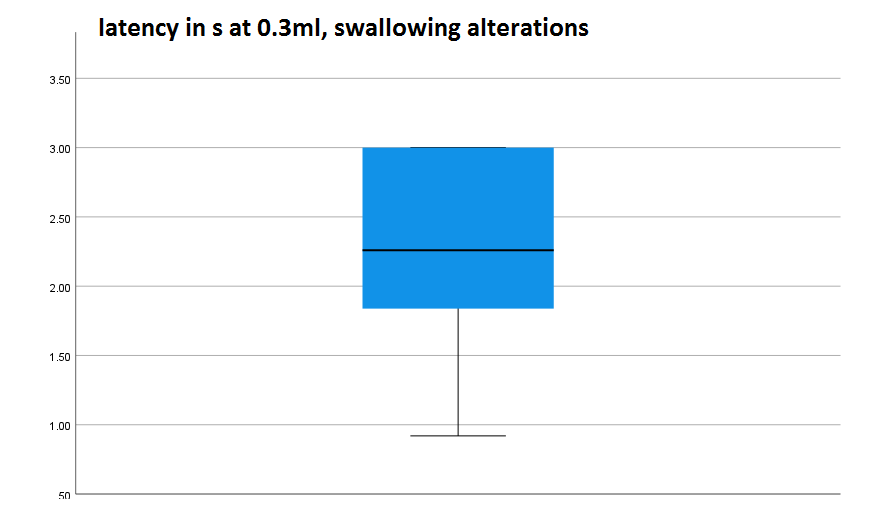


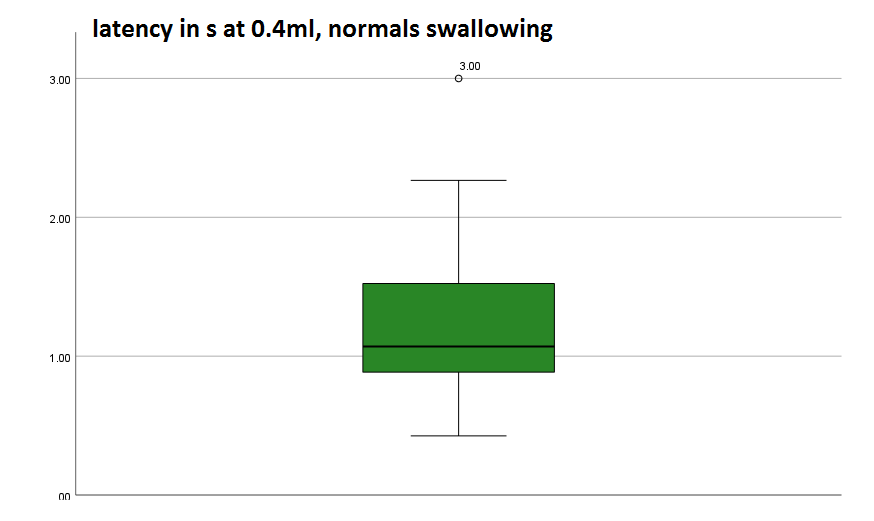


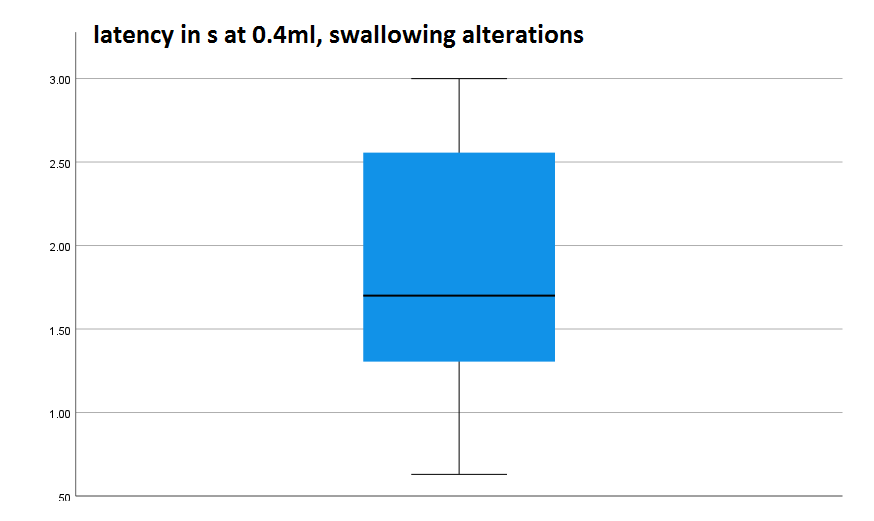


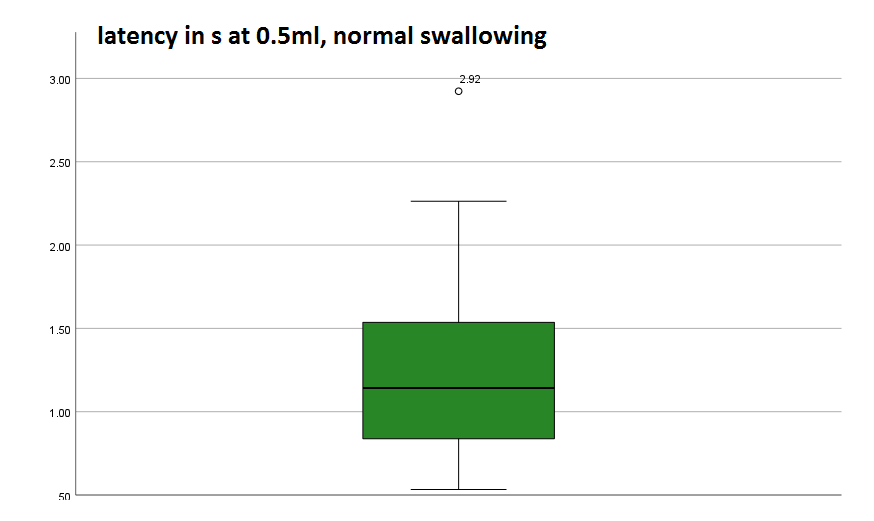


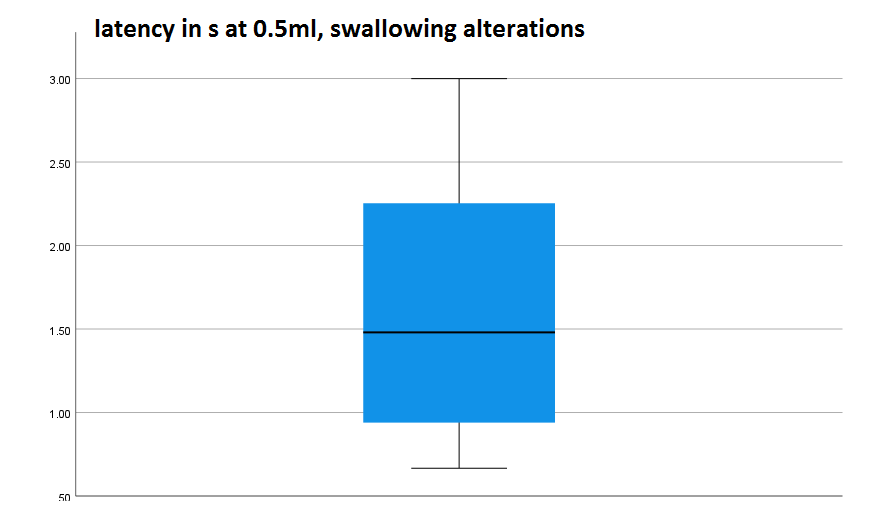


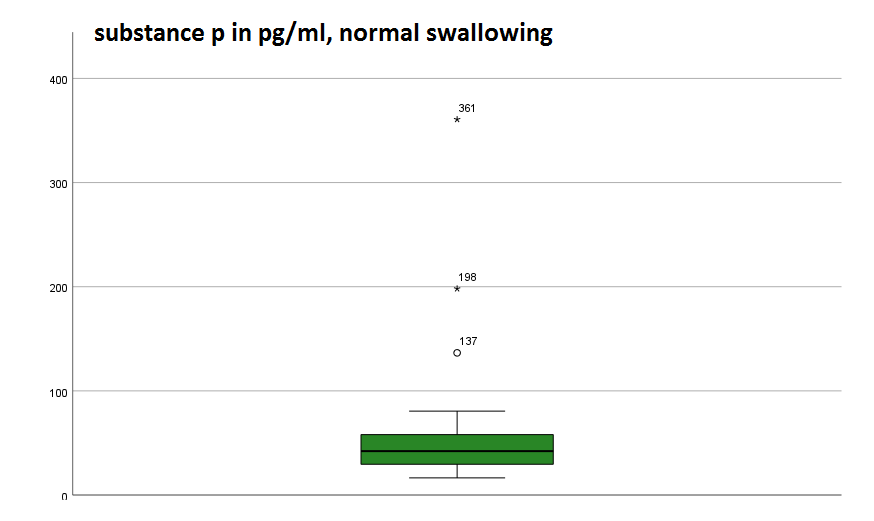


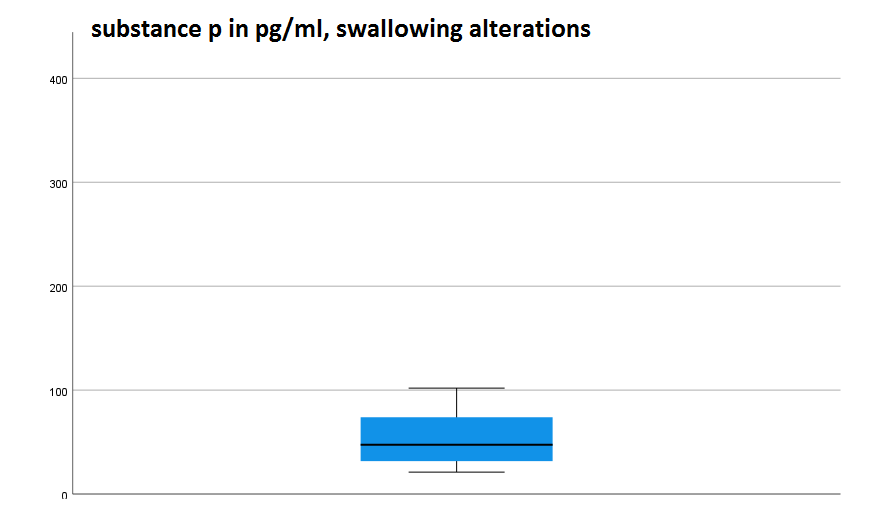


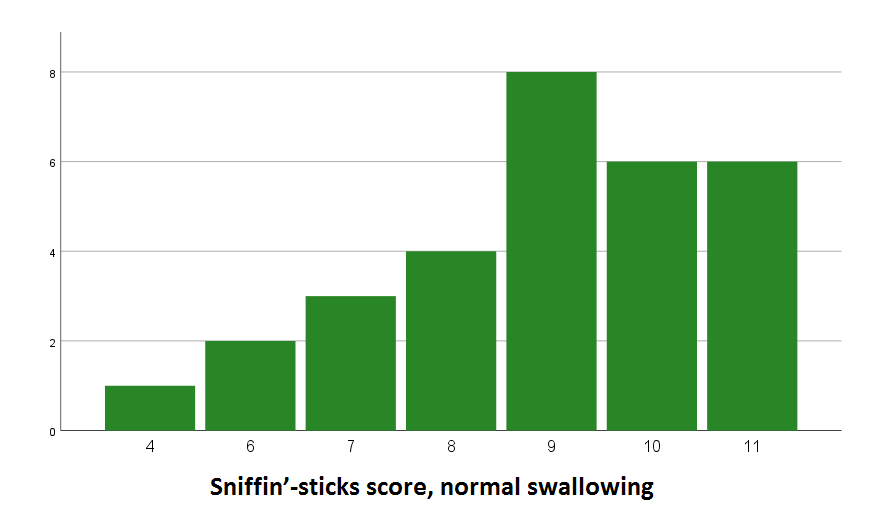


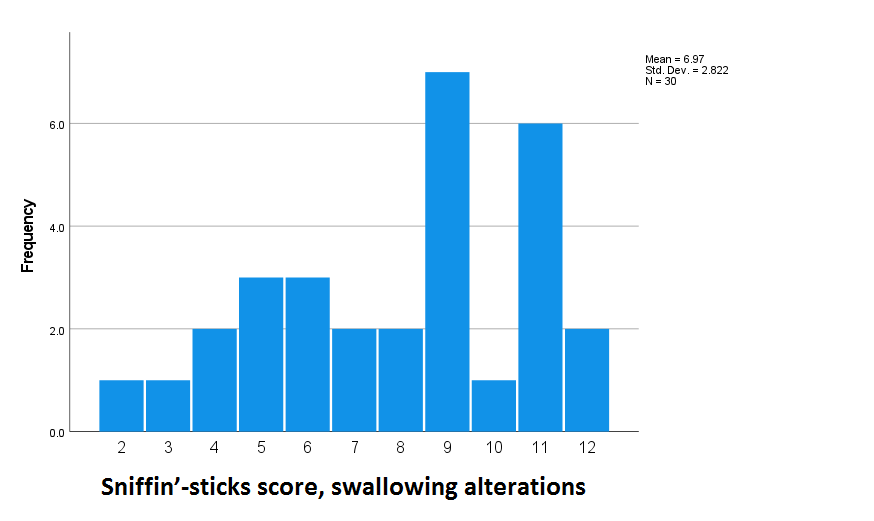


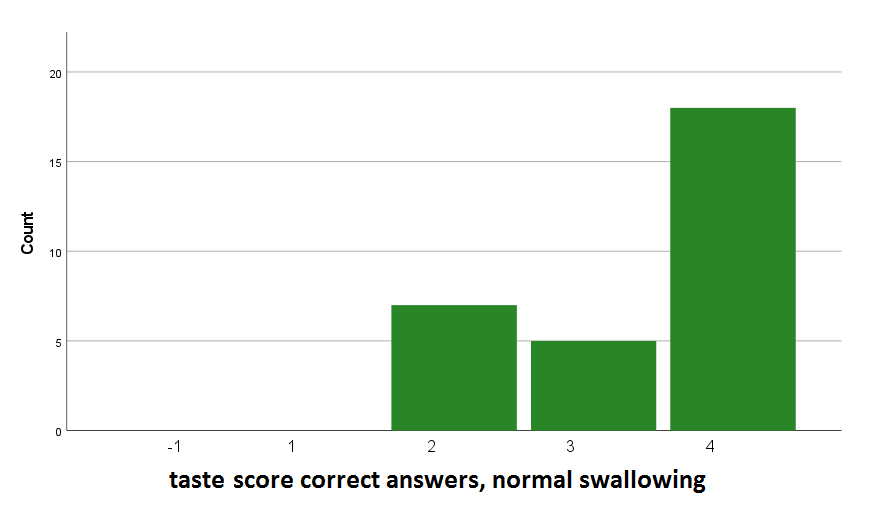


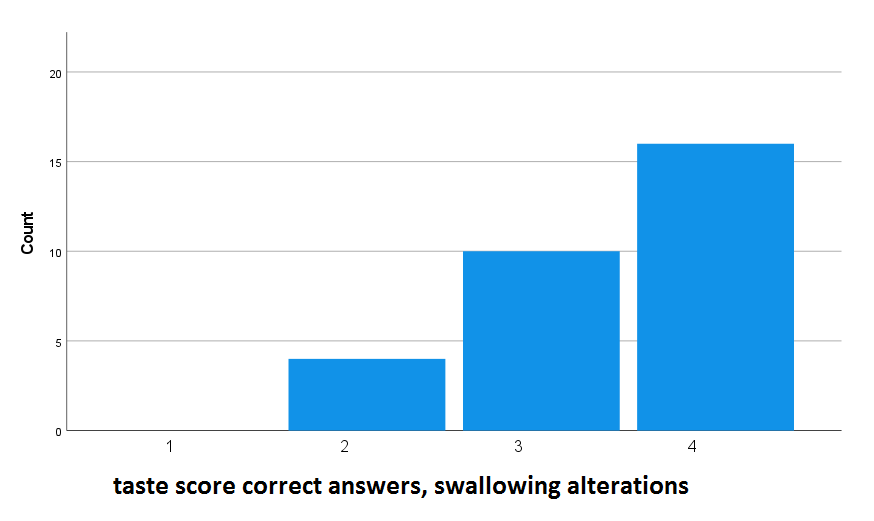


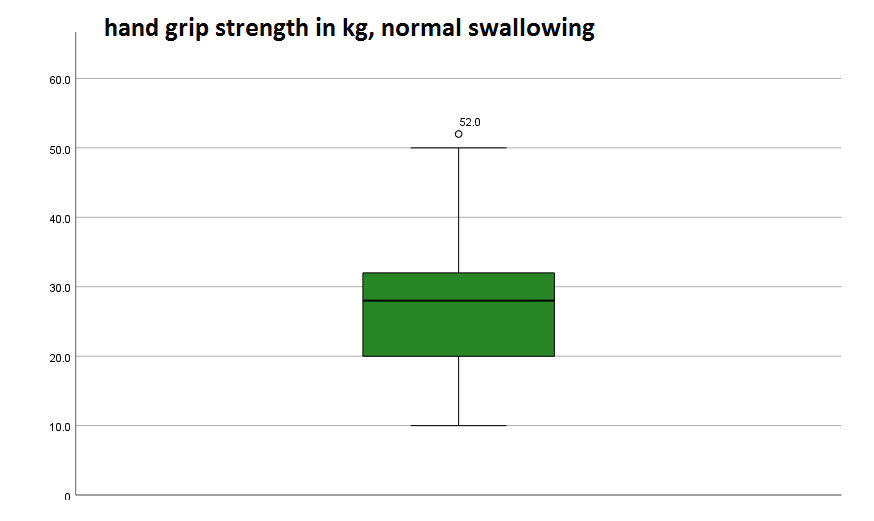


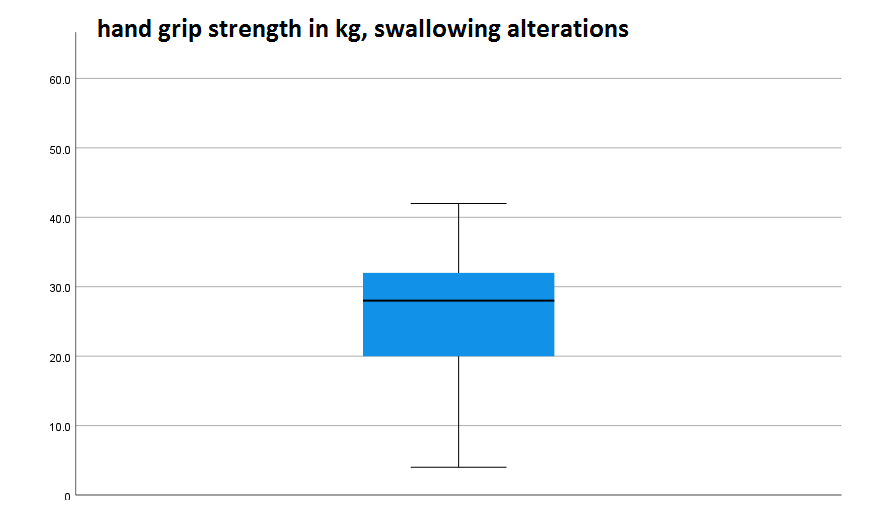


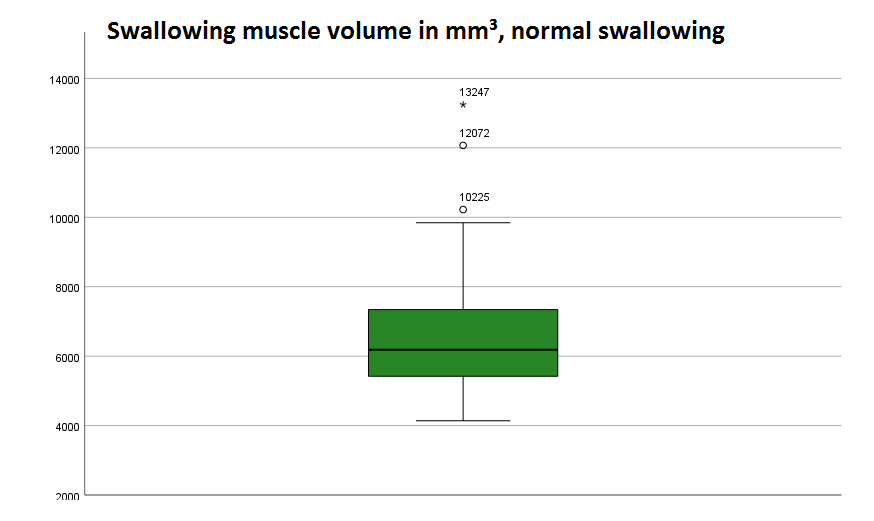


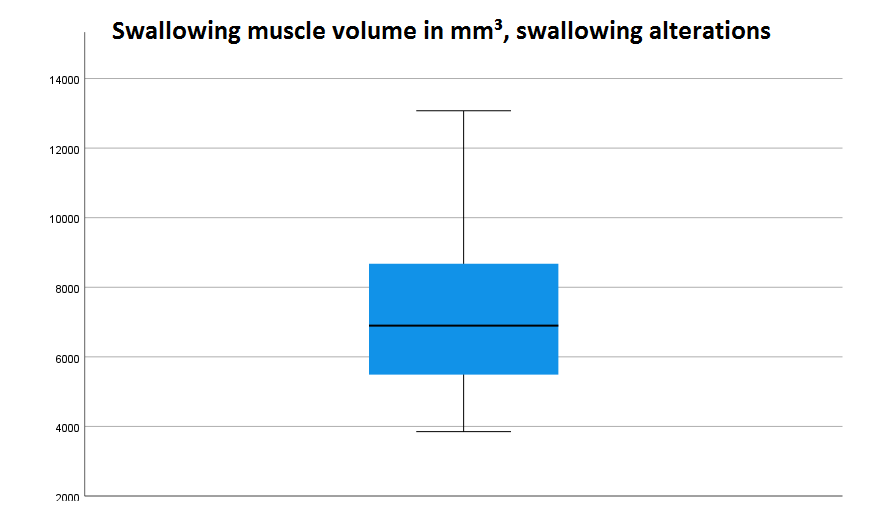


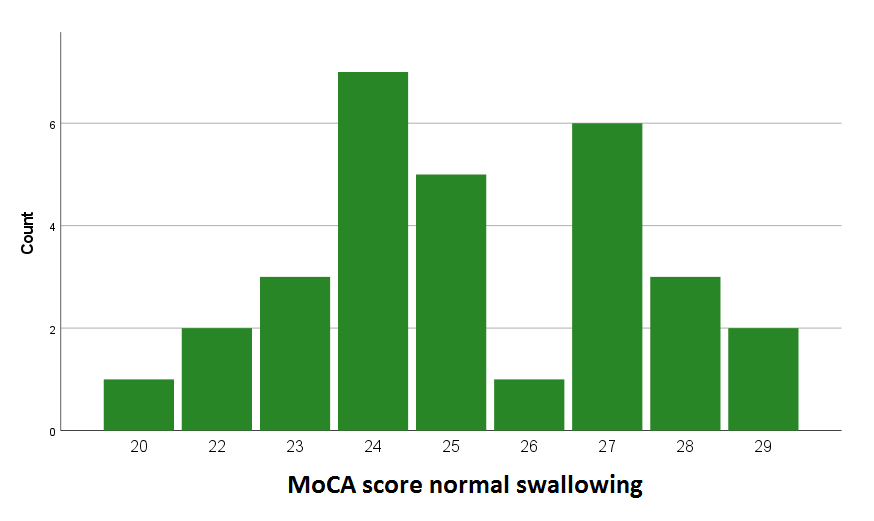


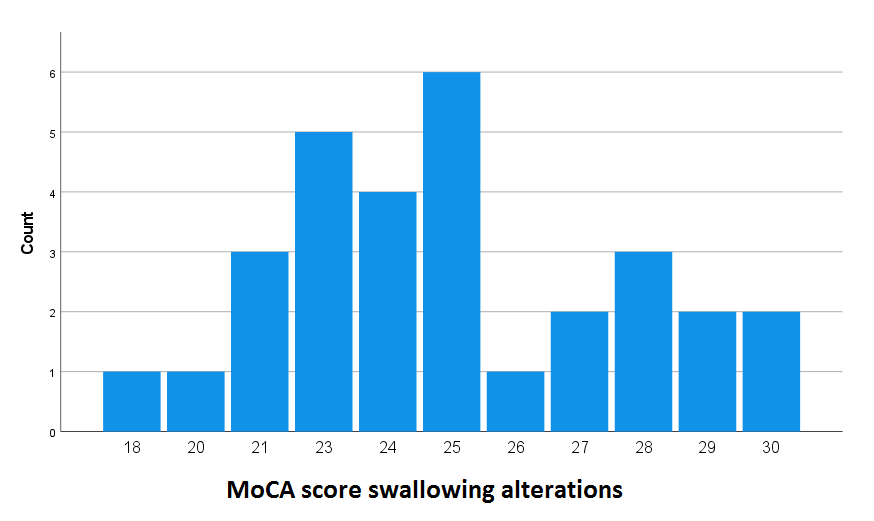


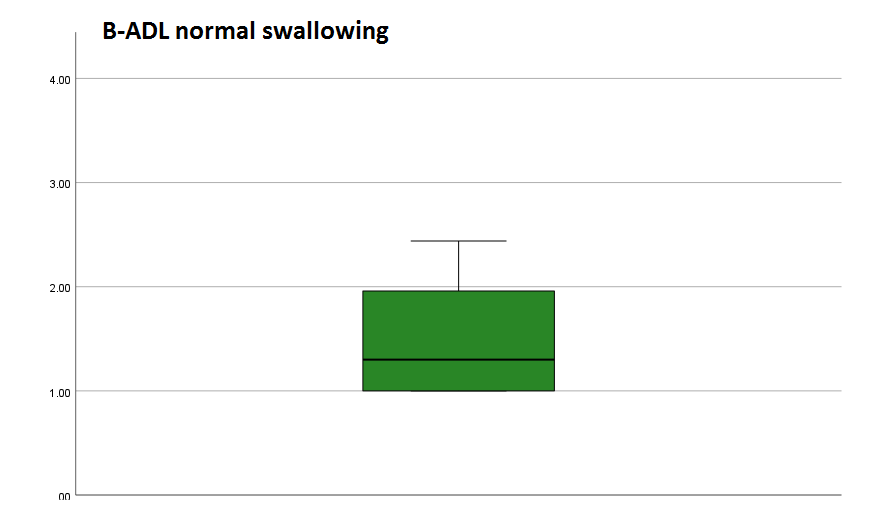


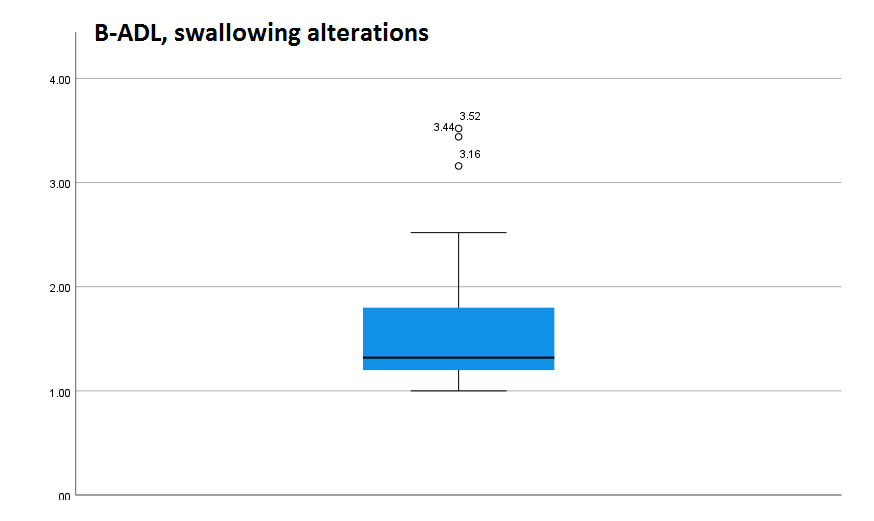


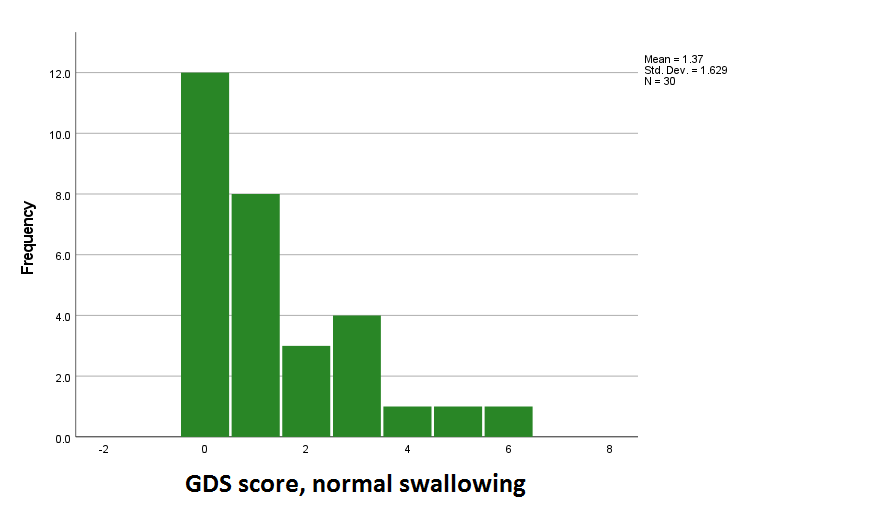


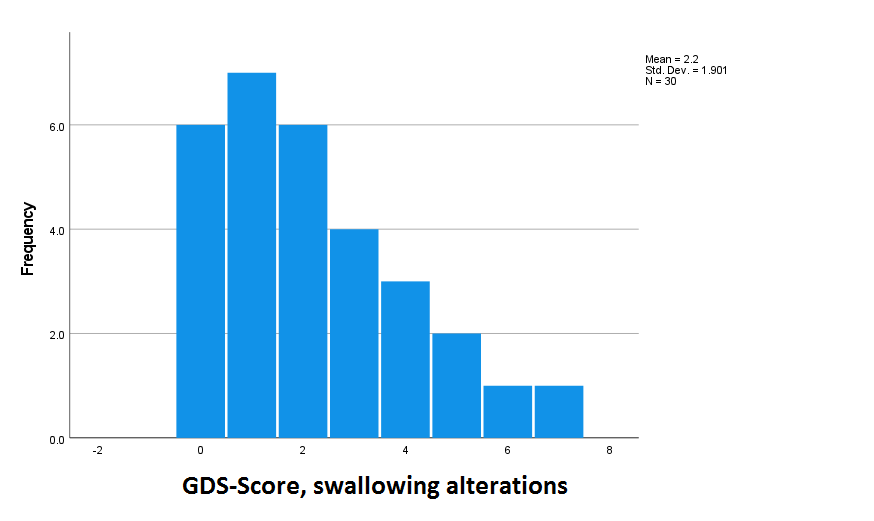


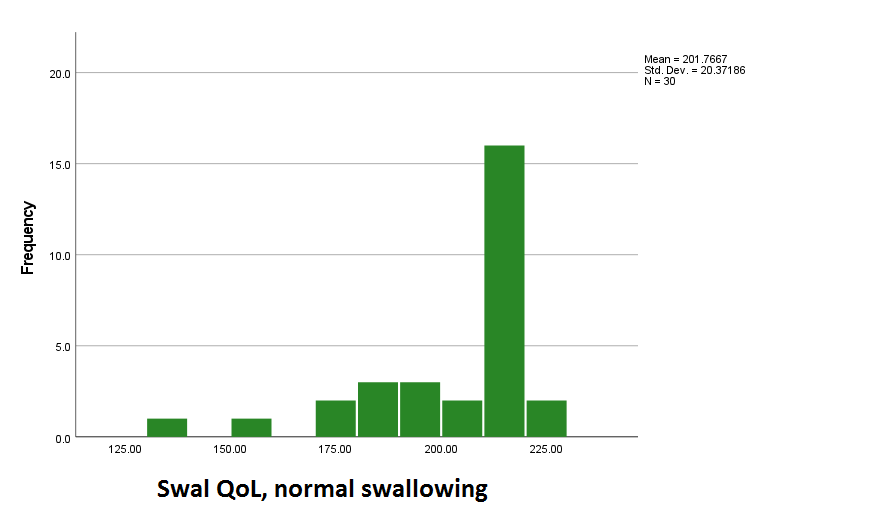


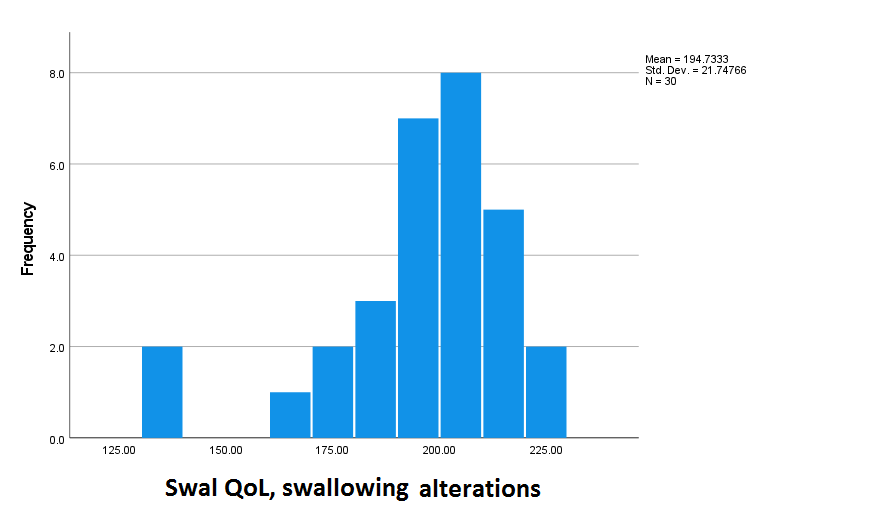


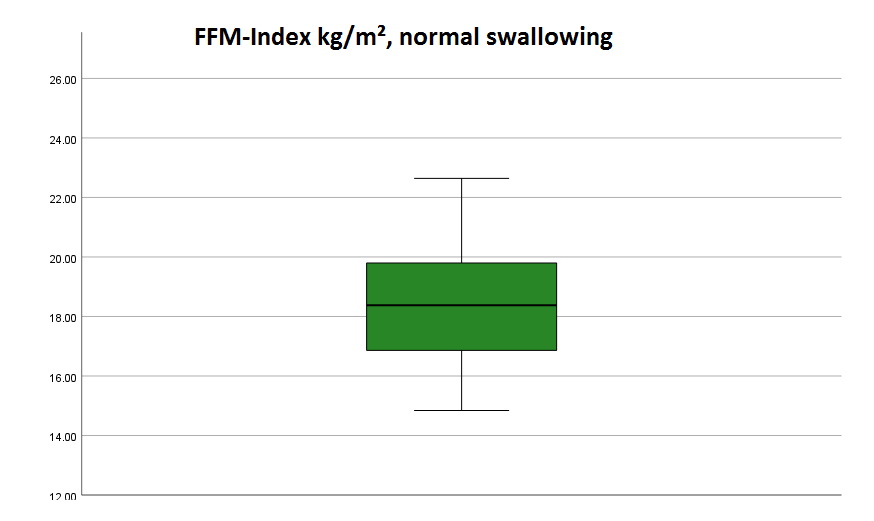


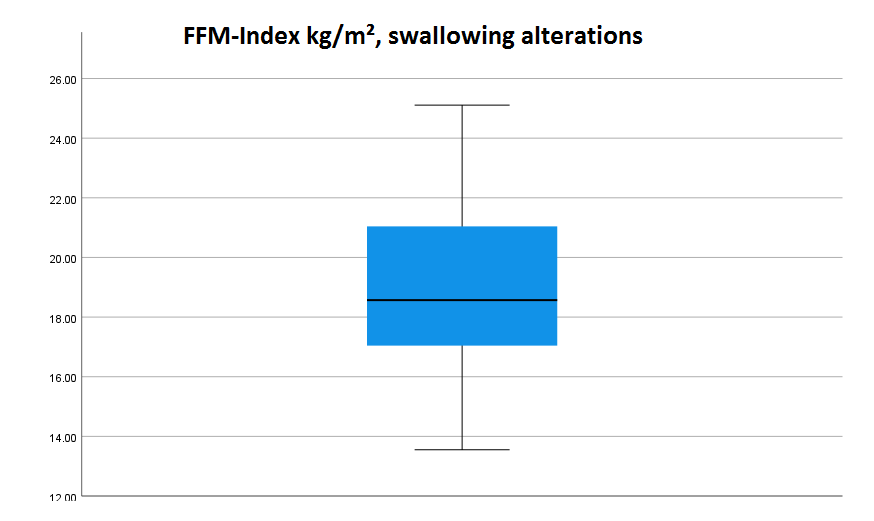


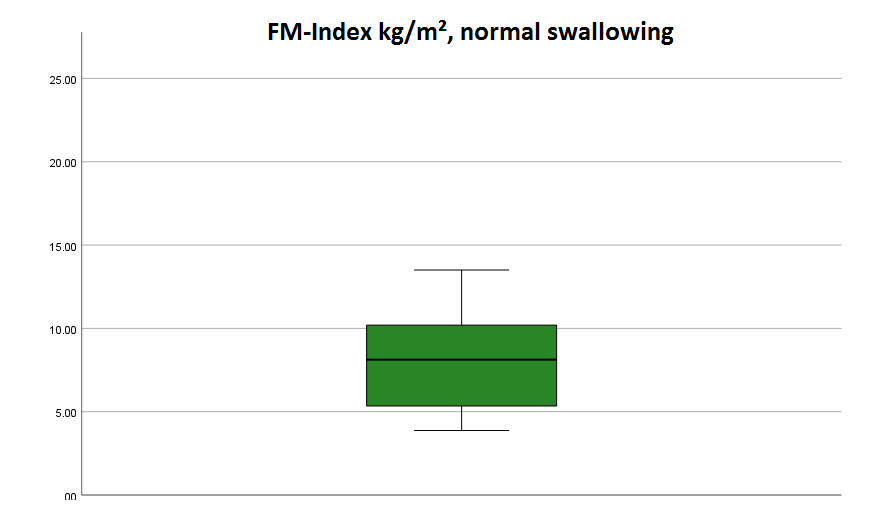


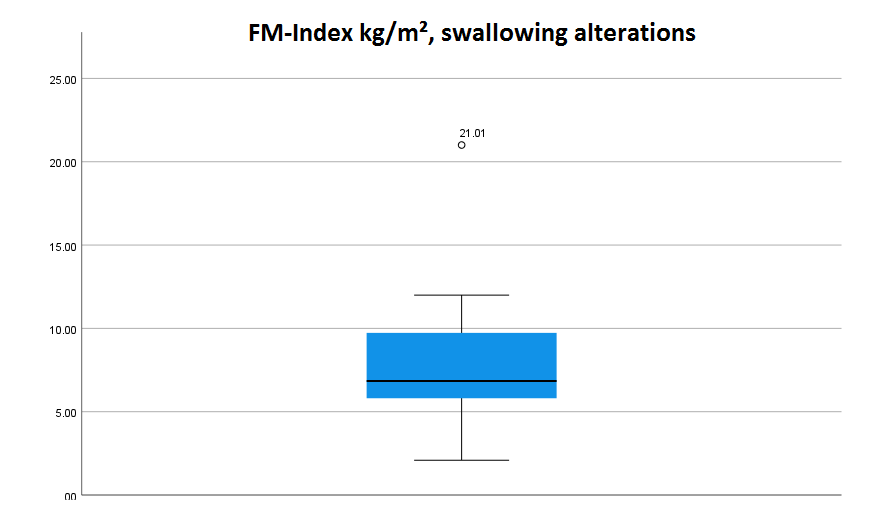


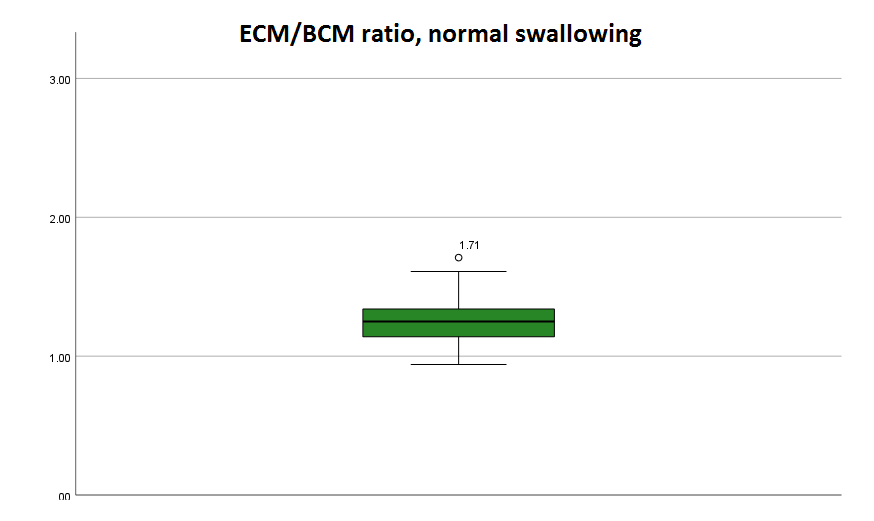


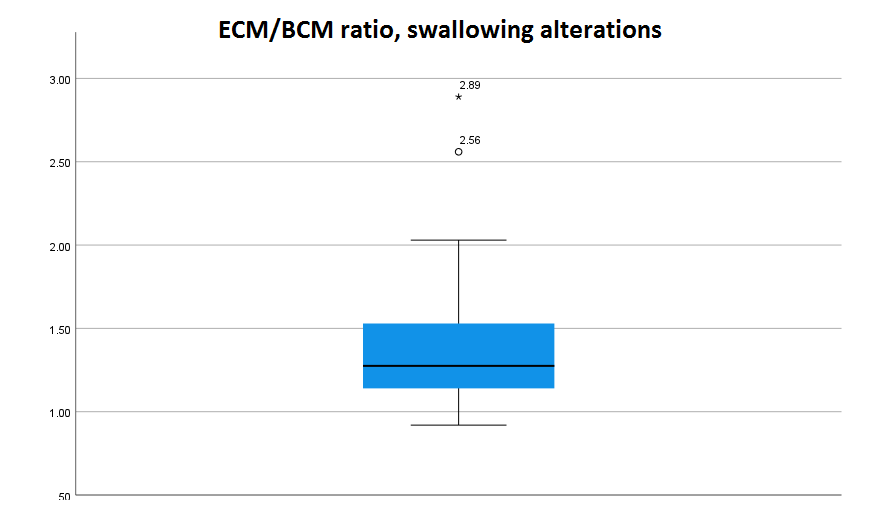


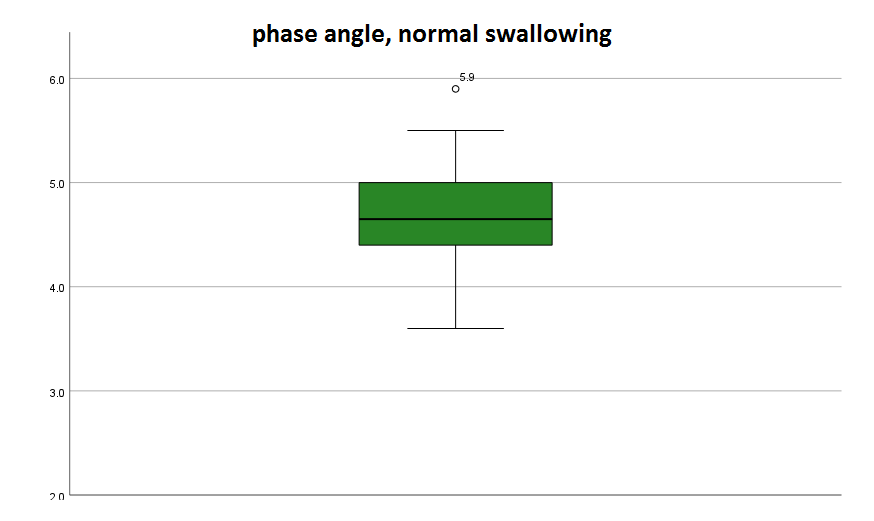


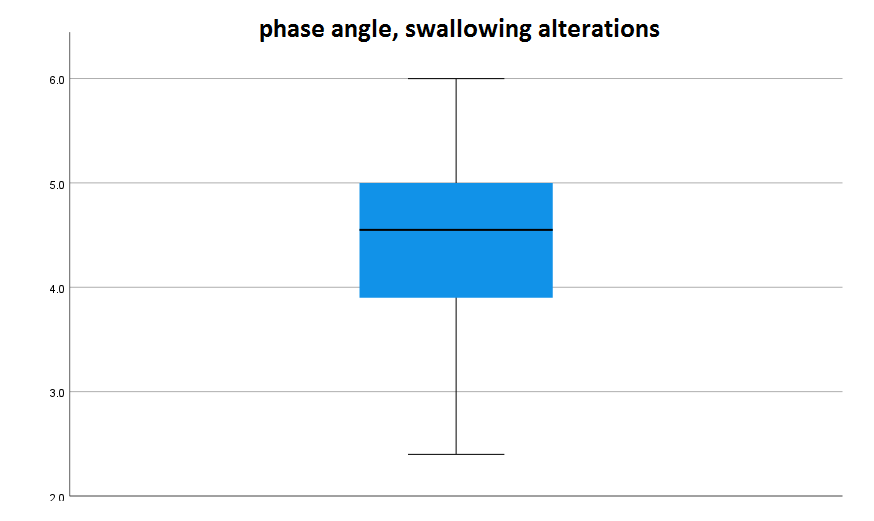


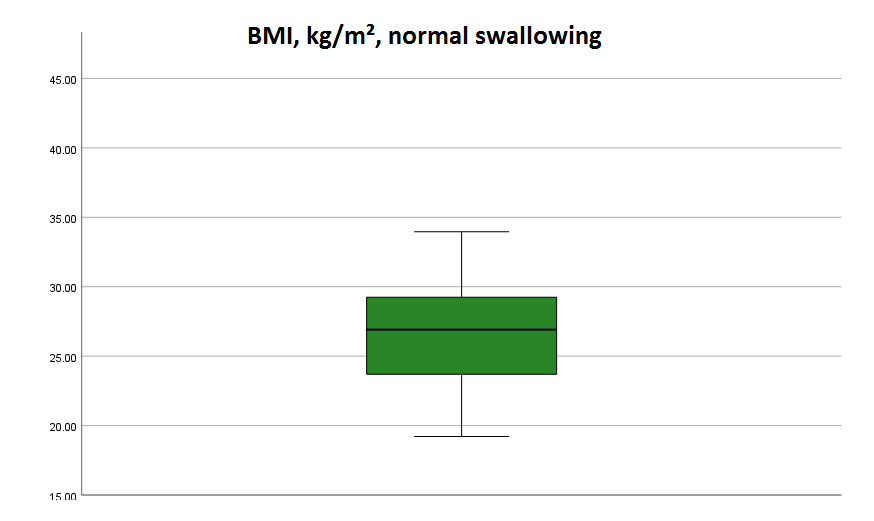


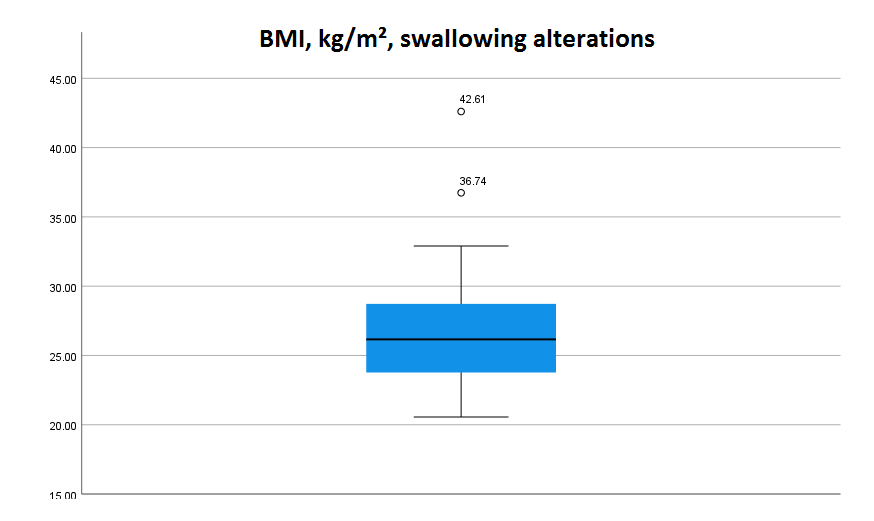


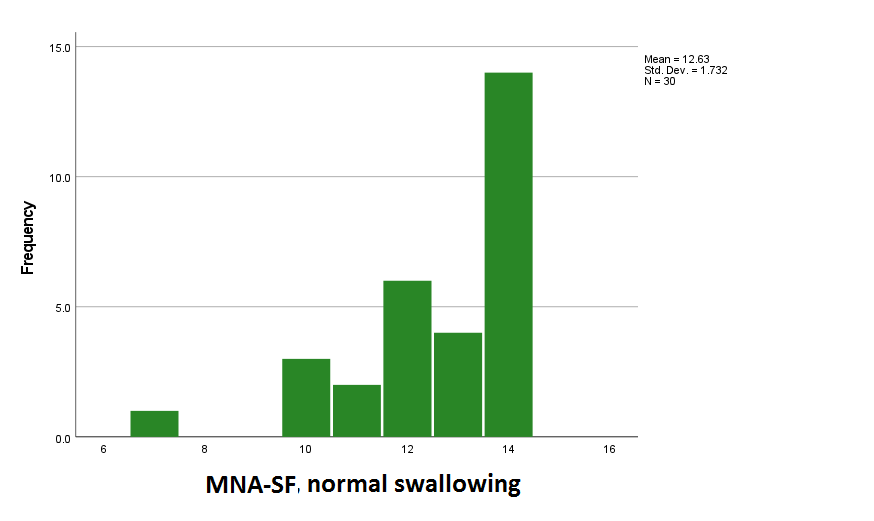


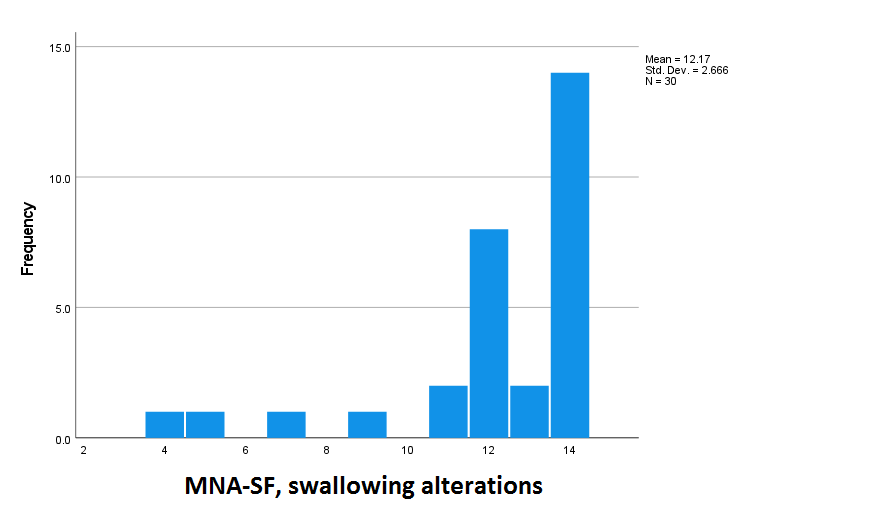


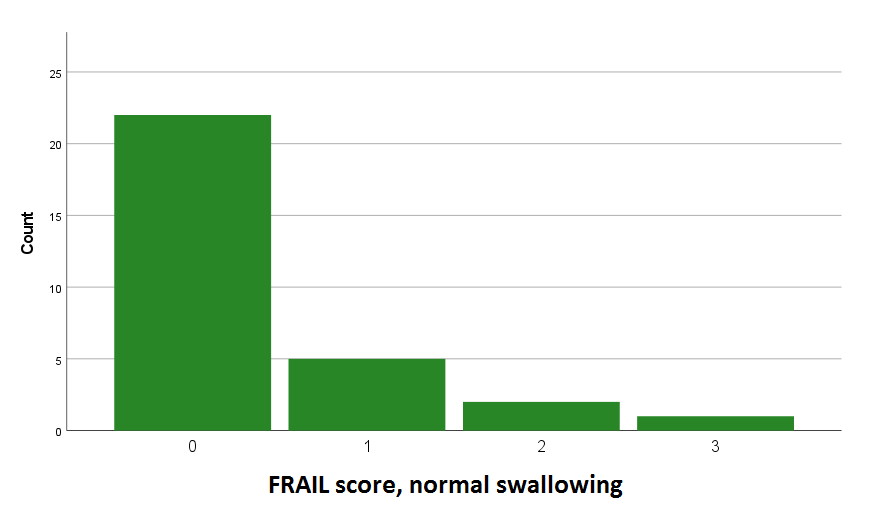


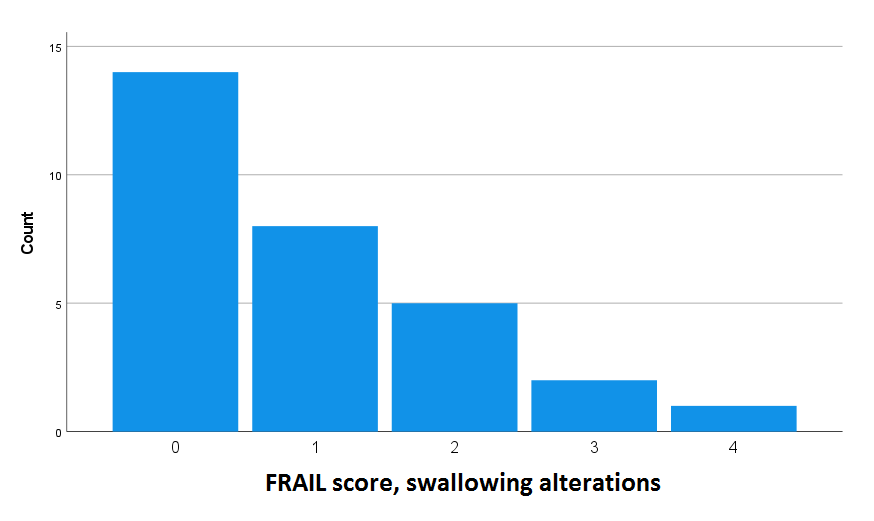

Supplement: Supplementary file 1 [file Data_Sheet_1.docx]
